# Personalised decision making to predict absolute metastatic risk in cutaneous squamous cell carcinoma: development and validation of a clinico-pathological model

**DOI:** 10.1016/j.eclinm.2023.102150

**Published:** 2023-08-19

**Authors:** Barbara Rentroia-Pacheco, Selin Tokez, Edo M. Bramer, Zoe C. Venables, Harmen J.G. van de Werken, Domenico Bellomo, David van Klaveren, Antien L. Mooyaart, Loes M. Hollestein, Marlies Wakkee

**Affiliations:** a Department of Dermatology, Erasmus MC Cancer Institute, Erasmus University Medical Center, Rotterdam, the Netherlands; b Department of Dermatology, Norfolk and Norwich University Hospital, Norwich, United Kingdom; c National Disease Registration Service, NHS England, United Kingdom; d Norwich Medical School, University of East Anglia, Norwich, United Kingdom; e Department of Immunology, Erasmus MC Cancer Institute, Erasmus University Medical Center, Rotterdam, the Netherlands; f SkylineDx B.V., Rotterdam, the Netherlands; g Department of Public Health, Center for Medical Decision Making, Erasmus University Medical Center, Rotterdam, the Netherlands; h Department of Pathology, Erasmus University Medical Center, Rotterdam, the Netherlands; i Department of Research, Netherlands Comprehensive Cancer Organization (IKNL), Utrecht, the Netherlands

**Keywords:** Cutaneous squamous cell carcinoma, Metastasis, Prognostic model, Absolute risk, Personalised medicine, Clinical decision support

## Abstract

**Background:**

Cutaneous squamous cell carcinoma (cSCC) is a common skin cancer, affecting more than 2 million people worldwide yearly and metastasising in 2–5% of patients. However, current clinical staging systems do not provide estimates of absolute metastatic risk, hence missing the opportunity for more personalised treatment advice. We aimed to develop a clinico-pathological model that predicts the probability of metastasis in patients with cSCC.

**Methods:**

Nationwide cohorts from (1) all patients with a first primary cSCC in The Netherlands in 2007–2008 and (2) all patients with a cSCC in 2013–2015 in England were used to derive nested case–control cohorts. Pathology records of primary cSCCs that originated a loco-regional or distant metastasis were identified, and these cSCCs were matched to primary cSCCs of controls without metastasis (1:1 ratio). The model was developed on the Dutch cohort (n = 390) using a weighted Cox regression model with backward selection and validated on the English cohort (n = 696). Model performance was assessed using weighted versions of the C-index, calibration metrics, and decision curve analysis; and compared to the Brigham and Women's Hospital (BWH) and the American Joint Committee on Cancer (AJCC) staging systems. Members of the multidisciplinary Skin Cancer Outcomes (SCOUT) consortium were surveyed to interpret metastatic risk cutoffs in a clinical context.

**Findings:**

Eight out of eleven clinico-pathological variables were selected. The model showed good discriminative ability, with an optimism-corrected C-index of 0.80 (95% Confidence interval (CI) 0.75–0.85) in the development cohort and a C-index of 0.84 (95% CI 0.81–0.87) in the validation cohort. Model predictions were well-calibrated: the calibration slope was 0.96 (95% CI 0.76–1.16) in the validation cohort. Decision curve analysis showed improved net benefit compared to current staging systems, particularly for thresholds relevant for decisions on follow-up and adjuvant treatment. The model is available as an online web-based calculator (https://emc-dermatology.shinyapps.io/cscc-abs-met-risk/).

**Interpretation:**

This validated model assigns personalised metastatic risk predictions to patients with cSCC, using routinely reported histological and patient-specific risk factors. The model can empower clinicians and healthcare systems in identifying patients with high-risk cSCC and offering personalised care/treatment and follow-up. Use of the model for clinical decision-making in different patient populations must be further investigated.

**Funding:**

PPP Allowance made available by 10.13039/100016036Health-Holland, 10.13039/100016036Top Sector Life Sciences & Health, to stimulate public-private partnerships.

**Research in context**

**Evidence before this study**

Cutaneous squamous cell carcinoma (cSCC) is the second most common skin cancer, causing an overall death toll comparable to that of highly studied aggressive cancers. Current clinical staging systems miss patients at high risk of metastasis; and they do not provide estimates of absolute metastatic risk. Moreover, the identification of risk factors has been based to date on single-centre retrospective cohorts with small numbers of metastatic cSCCs and non-representative distribution of risk factors and cSCC outcomes. The lack of accurate identification systems currently results in intensive long-term follow-ups recommendations for all patients with cSCC, causing a heavy burden on healthcare systems; and resulting in unnecessary anxiety for patients.

**Added value of this study**

We developed and externally validated - in two unique, fully characterised nationwide cohorts - an absolute risk model for predicting metastatic risk in patients with cSCC. The model was derived from a nested case-control cohort in the Netherlands, and subsequently validated in a large nested case-control cohort in England. The model has good discriminative ability in the validation cohort and is well-calibrated, outperforming or equating the staging systems for both metrics. More importantly, the model also ensures higher clinical utility than current staging systems, as assessed through decision curve analysis. To explore how metastatic risk estimates could be used for guiding patient care, the members of the multidisciplinary Skin Cancer Outcomes (SCOUT) consortium were surveyed to determine which metastatic risk cutoffs they would find appropriate to include a cSCC patient in a follow-up schedule; or to discuss alternative treatment options with their patients (adjuvant radiotherapy or adjuvant systemic treatment). We have also made the model available as a web-based calculator to facilitate model adoption.

**Implications of all the available evidence**

The absolute risk model that we developed for predicting metastatic risk in patients with cSCC has the potential to help clinicians in making more personalised decisions about the follow-up schedule and treatment of their patients with cSCC, as well as help improving the management of healthcare resources. Furthermore, the establishment of consensus risk thresholds like in our survey, together with the availability of the model as a web application, could facilitate adoption into clinical practice.

## Introduction

Cutaneous squamous cell carcinoma (cSCC) is the second most common skin cancer, affecting more than two million people worldwide yearly and metastasising in 2%–5% of patients.^1^, ^2^, ^3^ Due to its high incidence, the absolute number of cSCC-related deaths is comparable to that of aggressive and highly studied cancers, such as melanoma.^4^ However, our knowledge regarding the progression of cSCC is limited compared to other cancers.^5^ Current staging systems (e.g., Brigham and Women's Hospital^6^ [BWH] and the American Joint Committee on Cancer 8th edition [AJCC]^7^) are insufficient to identify all high-risk patients^8^^,^^9^; consequently, intensive long-term follow-up is recommended for all patients with cSCC.^10^^,^^11^ Providing personalised estimates of absolute metastatic risk would be transformational in personalised cSCC care: it would enable stratifying high-risk patients for consideration of adjuvant therapies, closer follow-up and screening investigations, and low risk patients for safe discharge^8^^,^^9^; while also facilitating the management of healthcare resources,^12^ as observed in other cancers.^13^^,^^14^ However, developing absolute risk models requires access to cohorts with representative incidence rates and distributions of risk factors, to allow correct estimation of metastatic risk,^15^ which is rare for cSCC.^16^ However, in the Netherlands, histologically confirmed cSCCs have been routinely registered in The Netherlands Cancer Registry (NCR) since 1989, and all cSCC pathology reports are available via linkage with the nationwide network and registry of histopathology and cytopathology (PALGA),^17^ allowing for estimation of accurate incidence of cSCC.^1^^,^^18^ Also, in England, all cSCC pathology reports are collected by the National Disease Registration Service with high quality data since 2013.^16^

Here, we integrated previous research on metastatic cSCC risk factors^19^ with the unique access to two full nationwide cSCC cohorts, to develop and validate a model that estimates the probability of metastasis in patients with cSCC.

## Methods

### Development cohort

From the Netherlands Cancer Registry (NCR), we selected all patients with a histopathologically confirmed first primary cSCC in 2007 or 2008 (12,325 patients). All patients were linked to the nationwide network and registry of histo- and cytopathology (PALGA)^17^ for retrieval of subsequent and metastatic cSCCs up to 26 August 2020, an update of the cohort described in Tokez et al.^1^ Cases who developed metastasis were identified using a rule-based algorithm applied to the conclusions of pathology reports, followed by a subsequent manual review, as described in the methods section of Tokez et al.^1^ and in the Supplementary text. A nested case-control (NCC) study was conducted to characterise in detail the clinical and pathological variables of all cases and matched non-metastatic controls^19^ (Supplementary Figure S1A). Matching was based on pathology lab and follow-up time: each case with a metastatic event at a given time t was matched to a control with a follow-up time greater or equal to t, and from the same pathology lab. Out of 267 patients identified with metastasis, the following ones were excluded: patients with metastasis at baseline (n = 34), patients whose tumour blocks could not be re-assessed (n = 24), patients with a metastasis of unknown primary origin (n = 5), patients where no distinction could be made between primary cSCC or cutaneous metastasis (n = 1), and patients without cSCC upon revision (n = 8), leading to a total of 195 metastatic cases and 195 non-metastatic controls. From those case-controls sets, the full pathology reports and Formalin-Fixed Paraffin-Embedded (FFPE) blocks of the primary tumours were retrieved from the pathology archives. In addition, the datasets were linked to the Netherlands Organ Transplant Registry (NOTR), to retrieve data on whether patients received organ transplants. From the NCR, data on haematological malignancies were obtained, since patients with a haematological malignancy were previously shown to have higher metastatic risk.^1^

### Validation cohort

The model was validated in a subset of a previously described cohort,^8^^,^^20^ consisting of a NCC study derived from patients with a primary cSCC registered in the National Disease Registration Service in England, between January 1st 2013 and December 31st 2015. Matching was performed based on follow up time: each case with a metastatic event at a given time t was matched to a control with a follow-up time greater or equal to t. To guarantee a reasonable follow-up time, only patients with a primary cSCC in 2013 were considered for the NCC cohort. Patients with metastasis at baseline (n = 38) were excluded (Supplementary Figure S1B).

### Ethics

The development study was approved by the Medical Ethics Review Committee (MERC; METC in Dutch) of the Erasmus Medical Center (MEC-2020-0147), and the scientific and privacy committees of the NCR, PALGA and NOTR. Informed consent from all patients was not required. Use of the validation cohort^8^ did not require ethical approval nor informed consent, as per Section 251 of the National Health Service Act 2006.

### Statistical analysis

#### Sample size

Sample size was determined in a previous study, which aimed to calculate the cumulative incidence of metastasis.^1^ For different scenarios with a maximum width of 1% of the 95% confidence interval (CI), 1522 (cumulative incidence of 1%, 95% CI: 0.5–1.5%) to 7299 (cumulative incidence of 5%, 95% CI: 4.5-5.5%) patients were needed. Therefore, we included all patients from two calendar years. The cohort of 12,325 patients included 195 incident metastatic events, which would allow to spend 19–20 degrees of freedom for prediction modelling in our development cohort.^21^

#### Patient and tumour characteristics

In the development cohort, patient characteristics were extracted from the NCR. Clinical tumour diameter and location were derived from the full pathology reports. All other histopathological characteristics were re-assessed by a dermatopathologist blinded to the outcome, using haematoxylin and eosin (H&E) slides of the tumours. Some variables were further transformed in the modelling part (Supplementary Table S1). In the validation cohort, tumour characteristics were derived from the pathology reports as previously described.^19^ Median follow-up was computed with the reverse Kaplan–Meier approach using the *prodlim* R package (v2019.11.13).^22^ Differences between cases and controls were tested using the Wilcoxon signed rank test with continuity correction for continuous variables, and the McNemar's Chi-squared test for categorical variables. Both tests account for the paired nature of the datasets.

#### Outcome

Primary outcome of interest was the occurrence of metastasis, defined as the presence of cancer cells in lymph nodes (i.e., locoregional metastasis) or other organs (including skin) outside the primary tumour site (i.e., distant metastasis), within 5 years. Only histopathologically confirmed metastases were considered events. Time to event was determined from the date of diagnosis of the primary cSCC until the date of identification of metastasis for cases, and until the date of death or last known follow-up for controls.

#### Multiple imputation

Missing values were assumed to be missing at random, and were imputed using multiple imputation using chained equations, using the *mice* R package (v3.13.0).^23^ Coefficients were pooled using Rubin's rules.^24^ Further details are described in the Supplementary Text.

#### Model development

A weighted Cox regression model^25^ with Kaplan Meier type weights^26^ was used to predict the probability of metastasis based on eleven routinely available clinico-pathological characteristics. The sampling weights in the model adjust for the difference in the ratio of cases to controls between the NCC dataset and the full cohort, and for the matching, so as to correctly estimate the absolute risk probabilities.^26^ Computation of sampling weights is described in the Supplementary Text.

To reduce the number of predictors in the final model, while keeping predictive ability, backward selection with the Akaike Information Criteria (AIC) was used. Follow-up was truncated at five years and numerical variables were centred. Proportionality assumption was checked with the *cox.zph* function of the *survival* package (v.3.3.1).^27^ Restricted cubic splines (from the *rms* package, v.6.2.0) were used to study potential non-linear relationships, but no significant non-linear relationship was detected. The final model was recalibrated, using uniform shrinkage based on bootstrap validation.^28^

We investigated the effect of competing events - death due to any cause - on the metastatic risk probability, but the effect was negligible (Supplementary Figure S2); therefore, it was not included in the final model.

#### Model evaluation

The model was internally validated using 100 bootstrapped samples. Discriminative ability was evaluated with the C-index.^29^ A value of 0.5 indicates no discrimination at all, whereas a value of 1 reflects perfect discrimination. The agreement between estimated and observed risks was assessed using the calibration slope and the observed to expected ratio (O/E ratio). The calibration slope corresponds to the slope of the regression of the survival outcomes on the model linear predictor.^30^ The O/E ratio corresponds to the ratio of the observed survival fraction to the average predicted risk at a specific timepoint.^31^ Since the percentage of controls in the NCC dataset is not representative of the full cohort, weighted versions of these metrics were computed,^32^ using the previously described weights.

The BWH^6^ and AJCC8^7^ staging systems were evaluated in both cohorts. For the BWH staging system, risk probabilities for nodal metastasis for each T-category are available^6^ and were used for calibration evaluation. This information is not available for the AJCC8 staging system.^7^ C-indexes of staging systems were compared to that of the absolute risk model using the nonparametric test proposed by Kang et al. for the comparison of C-indexes.^33^

We used decision curve analysis to assess the added clinical benefit of the model, relative to the default strategies of following-up all patients (“treat all”), and not following-up any patient (“treat none”), as well as the BWH and the AJCC8 staging systems. Net benefit estimates were weighted using the sampling weights.

Analyses were performed in R (v4.1.1). Code is stored in https://github.com/emc-dermatology/cSCC-abs-met-risk. Model development and validation was reported according to the TRIPOD guidelines (Supplementary Table S2).^34^

#### Model availability

Model predictions can be obtained using the formula in the supplementary information or calculated via a publicly available web application (https://emc-dermatology.shinyapps.io/cscc-abs-met-risk/).

#### Clinical survey

To explore how metastatic risk estimates could be used for guiding treatment decisions, 150 members of the Skin Cancer Outcomes (SCOUT) consortium were surveyed to determine which metastatic risk cutoffs they would find appropriate to 1) include a cSCC patient in a follow-up schedule, 2) discuss adjuvant radiotherapy to the local tumour bed following clear margin surgery with their patients with cSCC, and 3) discuss adjuvant systemic treatment with their patients with cSCC.

### Role of the funding source

The funder of the study had no role in the study design, data collection, analysis, interpretation, or writing of the report.

## Results

The model was developed in a NCC cohort with 390 patients diagnosed with a first cSCC in 2007/2008 in the Netherlands (195 metastatic cases and 195 non-metastatic controls), and externally validated in a NCC cohort of 696 patients diagnosed with a primary cSCC in 2013 in England (348 cases and 348 controls). In the cohort from the Netherlands, the cumulative incidence of metastasis at 5 years is 1.6% (1.4–1.8). In the cohort from England, the cumulative incidence of metastasis at 3 years is 1.5% (1.4–1.7). Controls included in the NCC cohorts are similar to the full nationwide cohorts (Table 1). The median follow-up time in the development cohort (9 years) is longer than in the validation cohort (2.4 years). In general, a higher proportion of cases had high risk clinico-pathological characteristics, but this difference was not reflected in current staging systems (Table 2). According to AJCC8, 39% (England) to 58% (Netherlands) of all metastatic cases were low-risk tumours (i.e., T1/T2) and according to BWH the percentage of low-risk tumours (T1/T2a) was 48% (England) to 71% (Netherlands).

**Table 1:** General characteristics of the full nationwide cohort from the Netherlands and the nested case–control (NCC) cohort derived from it (development cohort), as well as the full nationwide cohort from England and the NCC derived from it (validation cohort).

Characteristics	Development cohort (the Netherlands)			Validation cohort (England)		
	Full (N = 12,325)	Controls (n = 195)	Cases (n = 195)	Full (N = 31,981)	Controls (N = 348)	Cases (N = 348)
Median follow-up^a^ in years until metastatic event, death, or last date of follow-up, whichever comes first (IQR)	9.0 (3.9, 10.0)	8.1 (4.6, 12.4)	0.8 (0.4, 1.9)	2.4 (2.1,2.7)	2.52 (2.2, 2.8)	0.72 (0.3, 1.2)
Sex^b^, n (%)						
Female	5229 (42%)	78 (40%)	51 (26%)	11,286 (35%)	134 (39%)	71 (20%)
Male	7096 (58%)	117 (60%)	144 (74%)	20,695 (65%)	214 (61%)	277 (80%)
Median age^a^, years (IQR)	76 (67, 83)	76 (67, 80)	78 (70, 84)	80 (73,86)	80 (73, 87)	80 (72, 86)
Median number of cSCCs per patient, (IQR)	1 (1, 2)	1 (1, 2)	1 (1, 3)	1 (1,2)	1 (1, 2)	1 (1, 1)
Tumour location^a^, ^c^, n (%)						
Trunk and extremities	4114 (34%)	75 (39%)	31 (16%)	–	118 (34%)	79 (23%)
Scalp and neck	1557 (13%)	22 (11%)	25 (13%)	–	60 (17%)	73 (21%)
Face	6605 (54%)	98 (50%)	137 (71%)	–	167 (48%)	196 (56%)
Unknown	49	0	2	–	3	0

For each categorical variable, percentages of all variable levels in each column should sum up to 100% except for rounding differences.

Abbreviations: IQR Interquartile range.

a For the full cohorts, characteristics are measured at the diagnosis of first cSCC.

b Self-reported in the validation cohort.

c Tumour location could not be extracted for all records in the full nationwide cohort from England.

**Table 2:** Clinical and tumour characteristics of cases and controls of the nested case–control (NCC) cohorts from the Netherlands (development cohort), and from England (validation cohort).

Characteristics	Development cohort			Validation cohort		
	Controls (N = 195)	Cases (N = 195)	p-value^a^	Controls (N = 348)	Cases (N = 348)	p-value^a^
**Median Tumour diameter, cm (IQR)**	1.0 (0.7, 1.5)	1.6 (1.0, 2.5)	<0.001	1.2 (0.9, 1.7)	2.2 (1.5, 3.3)	<0.001
Unknown	50 (26%)	55 (28%)		16 (5%)	15 (4%)	
**Median Breslow thickness, mm (IQR)**	3.0 (2.0, 5.0)	4.5 (3.0, 6.0)	<0.001	3.0 (2.0, 4.5)	6.5 (4.3, 10.0)	<0.001
Undetermined^b^	2 (1%)	7 (4%)		0	0	
Unknown	0	0		38 (11%)	28 (8%)	
**Tissue involvement, n (%)**			<0.001			<0.001
Dermis	141 (73%)	64 (38%)		163 (73%)	89 (31%)	
Subcutaneous fat	39 (20%)	58 (34%)		52 (23%)	132 (45%)	
Beyond subcutaneous fat	12 (6%)	47 (28%)		7 (3%)	70 (24%)	
Undetermined^b^	3	26		0	0	
Unknown	0	0		126	57	
**Differentiation**^c^**, n (%)**			<0.001			<0.001
Good/Moderate	182 (93%)	145 (75%)		304 (89%)	175 (51%)	
Poor	13 (6.7%)	49 (25%)		36 (11%)	169 (49%)	
Undetermined^b^	0	1		0	0	
Unknown	0	0		8	4	
**Acantholytic or Desmoplastic or Spindle Morphology subgroup, n (%)**	13 (7%)	23 (12%)	0.11	11 (3%)	23 (7%)	0.06
**Perineural invasion**^d^**, n (%)**	9 (5%)	30 (15%)	0.001	15 (5%)	78 (26%)	<0.001
Undetermined^b^	0	10		0	0	
Unknown	0	0		47	49	
**Lymphovascular invasion, n (%)**	0 (0%)	7 (4%)	0.02	2 (1%)	53 (17%)	<0.001
Undetermined^b^	0	9		0	0	
Unknown	0	0		30	27	
**Resection margin, n (%)**			<0.001	–	–	–
Complete	163 (84%)	101 (53%)		–	–	
Incomplete	31 (16%)	90 (47%)		–	–	
Unknown	1	4		–	–	
**Tumour material, n (%)**			0.004	–	–	–
Biopsy	2 (1%)	15 (8%)		0 (0%)	0 (0%)	
Excision	193 (99%)	180 (92%)		348 (100%)	348 (100%)	
**Co-occurring Haematological malignancy**^e^**, n (%)**	8 (4%)	13 (7%)	0.38	18 (5%)	26 (8%)	0.28
**Organ transplant recipient**^e^**, n (%)**	18 (9%)	17 (9%)	1.00	8 (2%)	10 (3%)	0.81
**Site of first metastasis, n (%)**			–			–
Neck/parotid space	NA	139 (71%)		NA	252 (72%)	
Armpit	NA	18 (9%)		NA	59 (17%)	
Groin	NA	6 (3%)		NA	36 (10%)	
Other lymph node locations^f^	NA	6 (3%)		NA	1 (0.3%)	
Cutaneous metastasis	NA	24 (12%)		NA	0 (0%)	
Distant metastasis	NA	2 (1%)		NA	0 (0%)	
**BWH staging, n (%)**			<0.001			<0.001
T1	105 (73%)	47 (37%)		127 (68%)	42 (17%)	
T2a	30 (21%)	43 (34%)		46 (25%)	77 (31%)	
T2b	7 (5%)	33 (26%)		13 (7%)	115 (46%)	
T3	1 (1%)	5 (4%)		0 (0%)	17 (7%)	
Unknown^g^	52	67		162	97	
**AJCC8 staging, n (%)**			<0.001			<0.001
T1	110 (77%)	56 (44%)		135 (75%)	62 (26%)	
T2	18 (13%)	18 (14%)		19 (11%)	31 (13%)	
T3	14 (10%)	52 (41%)		25 (14%)	139 (59%)	
T4	0 (0%)	1 (1%)		0 (0%)	5 (2%)	
Unknown^g^	53	68		169	111	

For each categorical variable, percentages of all variable levels in each column should sum up to 100% except for rounding differences.

Abbreviations: AJCC8: American Joint Committee on Cancer 8th edition, BWH: Brigham and Women's Hospital, NA: not applicable, IQR: Interquartile range.

a Wilcoxon signed rank test with continuity correction for continuous variables; McNemar's Chi-squared test for categorical variables.

b Undetermined: Could not be determined, even after re-assessment of the H&E slides.

c Good/moderate: 25%-100 well differentiated; Poor: <25% well differentiated.

d Perineural invasion of nerve of any size.

e At the time of the primary cSCC.

f Lymph nodes located in the upper arm, retro-auricular area, or unknown location.

g No stage was assigned if any of the variables required by the staging systems was missing.

### Model development and internal validation

A weighted Cox regression model was fitted to the development dataset to predict absolute metastatic risk. Eight out of eleven variables remained in the prediction model after AIC backward selection (Table 3), including variables that are not used in current staging systems: age, sex, and number of prior cSCCs. The estimated hazard ratios are stable across imputations and are comparable to the results we obtained for the complete case analysis (Supplementary Table S3). The model was internally validated and showed good discriminative ability, with an optimism-corrected C-index of 0.80 (95% CI 0.75–0.85) (Table 4). The calibration slope (0.84 (95% CI 0.63–1.04)) was used as shrinkage factor of the regression coefficients to improve calibration. The final hazard ratios after shrinkage are shown in Table 3.

**Table 3:** Hazard ratios of the prediction model, before and after uniform shrinkage. The latter can be used to predict risk probabilities within 1 year (using baseline survival = 0.987), 3 years (baseline survival = 0.976) and 5 years (baseline survival = 0.973).

Variables	Unit/categories	Hazard ratios before shrinkage (95% Confidence Interval)	Hazard ratios after shrinkage (95% Confidence Interval)
Age	Per decade (10 years)	1.36 (1.15–1.60)	1.29 (1.12–1.48)
Sex	Female	1.00 (reference)	1.00 (reference)
	Male	1.85 (1.27–2.69)	1.67 (1.22–2.28)
Number of prior cSCCs	Per cSCC	1.99 (1.78–2.22)	1.78 (1.62–1.94)
Tumour location	Trunk and extremities	1.00 (reference)	1.00 (reference)
	Scalp/neck	0.42 (0.22–0.80)	0.48 (0.28–0.83)
	Face	1.85 (1.13–3.03)	1.67 (1.11–2.53)
Tumour diameter	In cm	1.91 (1.60–2.28)	1.72 (1.48–1.99)
Tissue involvement	Dermis	1.00 (reference)	1.00 (reference)
	Subcutaneous fat	1.48 (1.00–2.17)	1.39 (1.01–1.91)
	Beyond subcutaneous fat	5.34 (3.16–9.04)	4.06 (2.61–6.31)
Differentiation	Good/moderate	1.00 (reference)	1.00 (reference)
	Poor/undifferentiated	4.71 (3.28–6.77)	3.66 (2.70–4.95)
Perineural or Lymphovascular invasion	Absent	1.00 (reference)	1.00 (reference)
	Present	2.61 (1.61–4.21)	2.23 (1.49–3.33)
Breslow thickness	In mm	n.s	n.s
Immunosuppression	Organ transplant receiver or haematological malignancy	n.s	n.s
Morphology type	Acantholytic/Desmoplastic/Spindle Versus none/other	n.s	n.s

See Supplementary Text for mathematical formula. Uniform shrinkage was based on bootstrap validation: the calibration slope was calculated during bootstrap validation with 100 repetitions, and thereafter it was used as shrinkage factor on all the regression coefficients to improve calibration in external cohorts. Variables not selected by the model are denoted as n.s.

**Table 4:** Weighted performance metrics on the development cohort (internal validation with bootstrap, 95% confidence intervals), and on the validation cohort (external validation, bootstrap 95% confidence intervals) for model performances at 3 and 5 years.

	Development cohort		Validation cohort		
	C-index	Calibration slope	C-index	Calibration slope	O/E ratio
**3-year**					
Absolute risk model	0.80 (0.75–0.84)	0.83 (0.64–1.03)	0.84 (0.81–0.87)	0.96 (0.76–1.16)	0.82 (0.58–1.06)
BWH	0.75 (0.70–0.79)	0.72 (0.46–0.89)	0.82 (0.79–0.85)	0.83 (0.68–0.97)	0.56 (0.40–0.71)
AJCC8	0.71 (0.65–0.76)	–	0.79 (0.75–0.82)	–	–
**5-year**					
Absolute risk model	0.80 (0.75–0.85)	0.84 (0.63–1.04)	–	–	–
BWH	0.74 (0.69–0.79)	0.72 (0.44–0.89)	–	–	–
AJCC8	0.70 (0.65–0.76)	–	–	–	–

AJCC8: American Joint Committee on Cancer 8th edition staging system, BWH: Brigham and Women's Hospital staging system; O/E ratio: observed to expected events ratio.

### Model performance

The final model can be used to predict metastatic risk at different time points. Supplementary Figure S3 shows the distribution of estimated metastatic risk probabilities at 5 years for cases and controls. Risk probabilities, as expected, increased with increasing BWH stages, however, patients within each stage still showed a large variability in metastatic risk (Supplementary Figure S4).

**Fig. 1:**
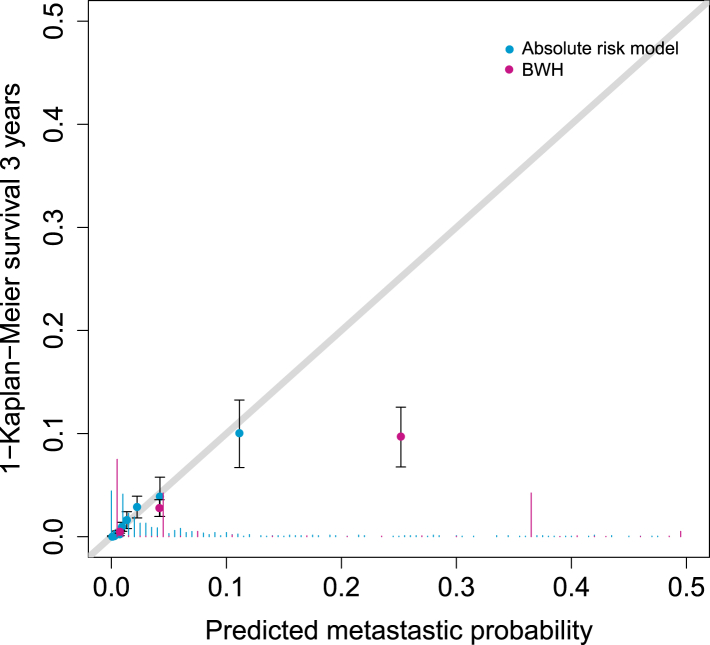
**Calibration plot.** Calibration plot comparing observed metastatic events in the validation cohort with metastatic events predicted by the absolute risk model (in blue) and the Brigham Women’s Hospital (BWH) staging (in pink). The histogram on the bottom shows the distribution of predicted risk probabilities. Patients were grouped into 10 groups of similar size (approximately 70 patients per group). Weighted average predicted metastatic probability and weighted Kaplan–Meier survival estimates were computed for each group. Confidence intervals correspond to the 95% confidence intervals of the Kaplan–Meier survival estimates for each group.

In the validation cohort, the C-index of our absolute risk model was 0.84 (95% CI 0.81–0.87) (Table 4), which is higher than that of the BWH and the AJCC8 (0.82 (95% CI 0.79–0.85, p-value = 0.09) and 0.79 (95% CI 0.75–0.82, p-value <0.01), respectively). The model was well-calibrated: the 3-year calibration slope was 0.96 (95% CI 0.76–1.16). The observed to expected ratio (O/E ratio) of 0.82 (95% CI 0.58–1.06) indicates that the model slightly overestimated metastatic events in this cohort, particularly for higher ranges of metastatic risk (Fig. 1). However, BWH staging is less well calibrated, with an O/E ratio of 0.56 (95% CI 0.40–0.71). Similar conclusions hold for performance metrics at 1 year (Supplementary Table S4) and for case–control sets without any missing data (Supplementary Table S5).

**Fig. 2:**
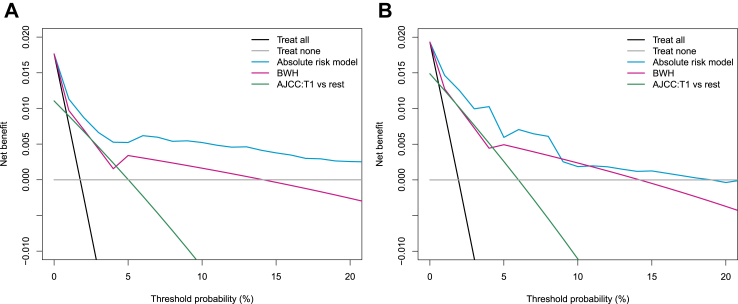
Decision curve analysis comparing the clinical utility of the absolute risk model and the Brigham and Women's Hospital (BWH) and American Joint Committee on Cancer 8th edition (AJCC8) staging systems on the (A) development cohort (from the Netherlands) and on the (B) validation cohort (from England). For the BWH system, risk probabilities for each T-category were used, as these are reported in the original publication.^6^ For the AJCC8 system, a decision cutoff was placed at T1 risk group versus remaining groups, since no risk probabilities are reported for each AJCC8 stage in the original publication.^7^ Net benefit estimates were weighted using sampling weights.

The clinical utility of the model was evaluated using decision curve analysis and was compared to current staging systems (Fig. 2). We focused on the range of probability thresholds between 0 and 20%, where missing a metastasis is at least 4 times worse than providing follow-up/treatment to a patient who will not develop a metastasis. This range covers the median decision cutoff that 53 surveyed experts would use for supporting decisions on a follow-up scheme for patients with cSCC (10% risk of metastasis) and on adjuvant radiotherapy (20% risk of metastasis), but not on adjuvant systemic treatment (30% risk of metastasis) (Supplementary Table S6). Decisions based on the absolute risk model led to higher net benefit compared to current staging systems in the 0–20% range, and outperform other default strategies (providing follow up/adjuvant treatment to everyone or to no one) in the 0–15% range.

## Discussion

In this study, we have developed and validated the first absolute metastatic risk prediction model in patients with cSCC, based on eight routinely available clinical and pathological characteristics. Despite the methodological challenge of predicting a rare event, model development and validation were possible due to access to two well-characterised population-based cohorts and a weighted approach for accurate estimation of metastatic risk and performance metrics. The model showed good discriminative ability, calibration, and clinical utility in the validation cohort, which is a promising development in the field of personalised medicine for patients with cSCC.

The pathological variables included in this absolute risk model are also present in AJCC8 or BWH staging systems. However, tumour diameter is continuous in our model, instead of being divided into categories as in the staging systems. This may prevent the loss of predictive information due to the categorisation.^35^ In addition, the remaining variables in the current model (age, sex, number of prior cSCCs and tumour location) are not included in AJCC8/BWH. However, studies have reported age-related differences in disease progression,^36^ as well as higher metastatic risk in males than in females.^20^^,^^37^ This can be due to a difference in ultraviolet (UV) exposure due to behavioural and physical differences between males and females, as well as sex-related differences related to the immune system.^37^ The number of prior cSCC tumours has also been associated with increased metastatic risk,^38^ and might serve as a proxy for other factors: sun exposure and immunosuppression status, both linked to the development or aggressiveness of cSCCs.^39^^,^^40^ Tumour location is also used as an additional risk stratification factor in the cSCC NCCN guidelines.^11^ The impact of tumour location might reflect a direct etiological explanation by its anatomical position but could also be related to more narrow excision margins that are applied in practice in cosmetically more challenging locations. Surprisingly, Breslow/tumour thickness was not included in the model, which might have been because its predictive effect has been partially captured by structural invasion and tumour diameter. Similarly, immunosuppression was not selected for the final model, which is in line with findings of other groups that immune suppression is not predictive of poor outcomes in cSCC after adjusting for tumour characteristics.^41^

Compared to the AJCC8 and BWH staging systems, which cluster patients into heterogenous risk groups (as shown in Supplementary Figure S4), our absolute risk model has better discriminative ability and provides well-calibrated personalised metastatic risk estimates for different time points (1, 3 and 5 years). More importantly, decision curve analyses showed that decisions based on our model were better or comparable to using the BWH staging system across different probability thresholds. Having access to reliable, personalised risk estimates of metastasis can have an important impact on medical decisions directly affecting patient related outcomes as well as healthcare systems. For example, high 1-year metastatic risk estimates could support decisions on adjuvant radiotherapy, while low 5-year metastatic risk estimates could help decrease surveillance intensity. Furthermore, many cSCC guidelines struggle with integrating staging systems and other known cSCC risk factors, resulting in additional flow-charts indicating different combinations of risk factors for different medical decisions to identify patients with high-risk cSCC that would benefit from each procedure,^11^^,^^42^^,^^43^ complicating the decision process. Our model incorporates such prior knowledge into a unified, easily accessible model and provides risk estimates at different timepoints, which can be particularly helpful to support decision making, especially for the often older and frail patients with cSCC. While setting a universal cutoff on the model output is tempting, the optimal cutoff depends on how acceptable it is to refer patients to a treatment to prevent one metastatic event, which in turn depends on the impact per treatment, available healthcare resources and patient characteristics.

A strength of this study is the thorough data collection of the development cohort: it was based on a large nationwide cohort with 10 years of follow-up. Moreover, all slides were reanalysed by a dermatopathologist to ensure a consistent assessment of pathological characteristics and to guarantee a low percentage of missing data during model development. Model validation on a large and geographically distinct cohort provides strong evidence of the generalisability of the model, especially considering that all variables were extracted from routine pathology reports without any reassessment of histologic slides; and the number of previous cSCCs was underestimated, because complete access to patient cSCC records was only possible in a limited time period (2013–2015). Of note, the performance of our model and the staging systems was higher in this cohort, which might be due to the shorter follow-up time in this cohort, but can also indicate some bias into a more detailed scoring of high-risk clinico-pathological characteristics for cases (as pathological characteristics were retrospectively extracted from pathology reports, without any reassessment by a pathologist as was done for the development cohort). Moreover, the validation cohort only contains tumours with excision records (as there was minimal histological information in records of curettages and shave biopsies). Nevertheless, the head-to-head comparison between our model and the staging systems in this cohort is still informative and demonstrates the added clinical utility of our model relative to the staging systems.

This study also has some limitations. Although the sample size in the development cohort allowed for sufficient power to detect associations between predictors and metastatic events, this was only possible because some variables were grouped, such as the presence of perineural and lymphovascular invasion, and tumour locations. Also, not all metastatic events could be captured: some metastases were excluded because the primary tumour could not be reliably identified (e.g., in patients with multiple cSCCs), and only histopathologically confirmed metastasis were included as events. The latter might have led to missing distant metastases in case there has not been prior or concomitant nodal metastasis. However, since almost all distant metastases are preceded by lymph node metastasis, this proportion is expected to be low.^44^ Furthermore, the follow-up time in the validation cohort was too short to evaluate the model in predicting metastatic risk at 5 years. Nevertheless, the performance at 1 and 3-years could be successfully evaluated. Finally, the model slightly overestimated metastatic risk in the validation cohort. However, overestimation was mainly observed in the highest probability ranges. Therefore, the impact of this limitation is assumed to be small in clinical decision making. Of note, our absolute risk model was specifically designed to predict metastatic outcome, as the presence of metastasis is one of the main causes of death in patients with cSCC,^45^ and their early detection is expected to improve treatment related morbidity and survival of these patients.^46^ Therefore, it is worth identifying patients at higher risk of metastasis for increased surveillance. However, there are other risk outcomes in patients with cSCC that would be important to predict and could inform therapeutic choices or the intensity of surveillance of these patients, such as recurrent cSCC, development of multiple primaries, locally advanced cSCC and death due to cSCC.

The predictive ability of our absolute risk prediction model could be even further increased by incorporating other data types, for instance molecular data and whole slide image analysis, to identify aggressive tumours that do not display high-risk clinico-pathological features at first diagnosis. Despite their potential,^47^^,^^48^ further research is still needed to identify reliable molecular/imaging prognostic biomarkers.^49^

To conclude, the presented model provides estimates of metastatic risk for patients with cSCC, was validated in an independent cohort and is freely available as a web-based calculator (https://emc-dermatology.shinyapps.io/cscc-abs-met-risk/). We envision that the model can be used to improve management of patients with cSCC by supporting more balanced decisions on appropriate treatments and adapting follow-up schedules according to personalised metastatic risk estimates.

## Contributors

LH and MW conceived the study. BRP, LH, and MW designed the study. ST, EB, ZCV, and AM contributed to data collection and interpretation. BRP, ST and LH had direct access to and verified the data from the Netherlands, and BRP and ZV to the data from England. BRP did the statistical analysis and modelling work. BRP wrote the first draft of the manuscript. HvW, DB, DvK, LH, and MW supervised the study. All authors interpreted the data and critically reviewed the manuscript. All authors accept responsibility for the decision to submit this publication.

## Data sharing statement

Due to the different data-sharing policies of the various datasets included in this study, data included in this study will not be made available. Requests for the data from each included dataset should be made to The Netherlands Cancer Registry (NCR), the nationwide network and registry of histopathology and cytopathology (PALGA), and English National Disease Registration Service.

## Declaration of interests

MW participated as speaker/advisory board member/consultant for Sanofi Genzyme, Sunpharma and LEO Pharma. DB is employed by SkylineDx. BRP was employed by SkylineDx before beginning the submitted work (December 2019–July 2021). All other authors declare no competing interests.
