# Supplementary material for: Personalised decision making to predict absolute metastatic risk in cutaneous squamous cell carcinoma: development and validation of a clinico-pathological model
Source: eClinicalMedicine. 2023 Aug 19;63:102150. doi: 10.1016/j.eclinm.2023.102150 (PMC10468358; doi:10.1016/j.eclinm.2023.102150)
Supplement: Supplementary Material [file mmc1.docx]

**Supplementary Material**

Personalised decision making in cutaneous squamous cell carcinoma: development and validation of a clinico-pathological model to predict absolute metastatic risk

**Supplementary Figure 1** Consort diagrams for the development cohort from the Netherlands and the validation cohort from England 2

**Supplementary Figure 2** Investigation of the effect of competing events (in particular death due to any cause) on the estimated probability of metastasis. 2

**Supplementary Figure 3** Distribution of risk probabilities estimated by the absolute risk model, for cases and controls 3

**Supplementary Figure 4** Distribution of estimated metastatic risk probabilities within BWH stages and within AJCC8 stages, for the development cohort and the validation cohort. 3

[**Supplementary Table 1** Predictors considered for the model, how they were measured in the Dutch cohort and how they were recoded for model development. n.a: not applicable, NCR: National Cancer Registry from the Netherlands 4](#_Toc135822834)

[**Supplementary Table 2** Transparent reporting of a multivariable prediction model for individual prognosis or diagnosis (TRIPOD) checklist for prediction model development 6](#_Toc135822835)

[**Supplementary Table 3** Hazard ratios (before shrinkage) estimated in the development cohort considering complete cases 7](#_Toc135822836)

[**Supplementary Table 4** Weighted performance metrics on the development cohort (internal validation with bootstrap), and on the validation cohort (external validation, bootstrap confidence intervals) for model predictions at 1 year. 8](#_Toc135822837)

[**Supplementary Table 5** Weighted performance metrics on the validation cohort (external validation, bootstrap confidence intervals) for model predictions at 3 years. Only case-control sets without any missing variables for the absolute risk model/staging systems were considered (N=288). 8](#_Toc135822838)

[**Supplementary Table 6** Overview of profession and work location of the respondents to the survey (N=53), and their answers regarding metastatic risk cutoff for guiding decisions on follow-up schedule, adjuvant radiotherapy, and systemic treatment of cSCC patients.. 9](#_Toc135822839)


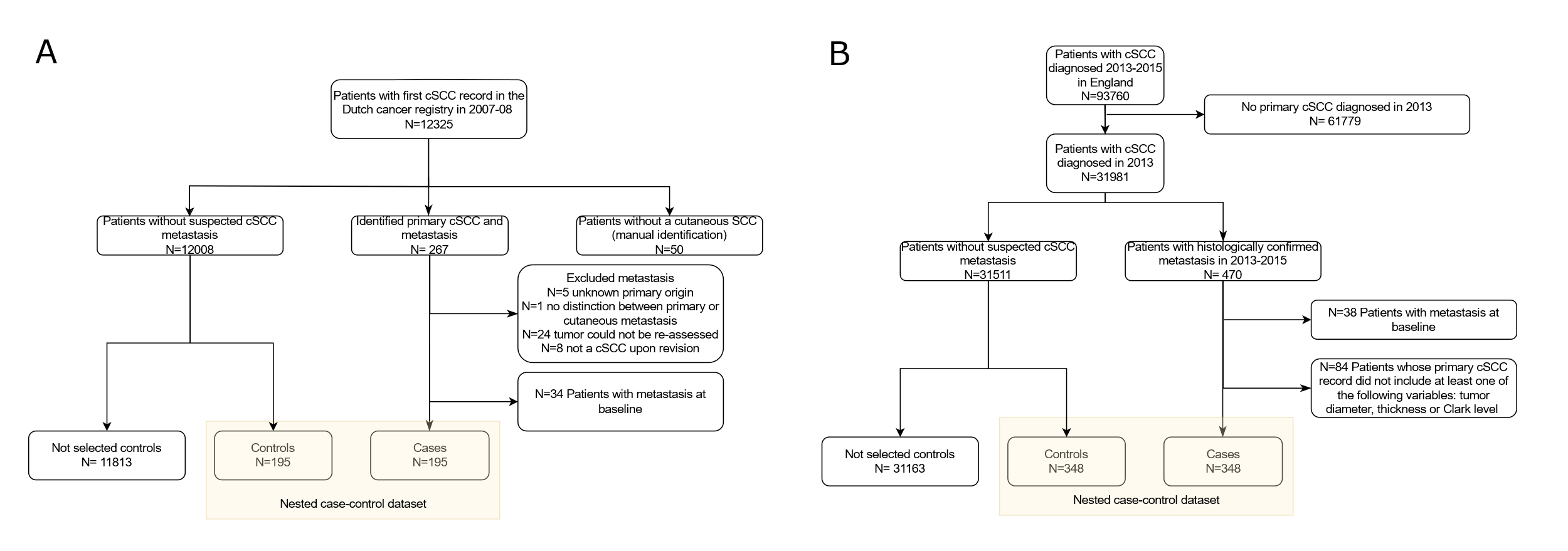


**Supplementary Figure 1** Consort diagrams for the development cohort from the Netherlands (A) and the validation cohort from England (B). N indicates number of patients. Yellow boxes indicate the patients in the final nested case-control dataset.


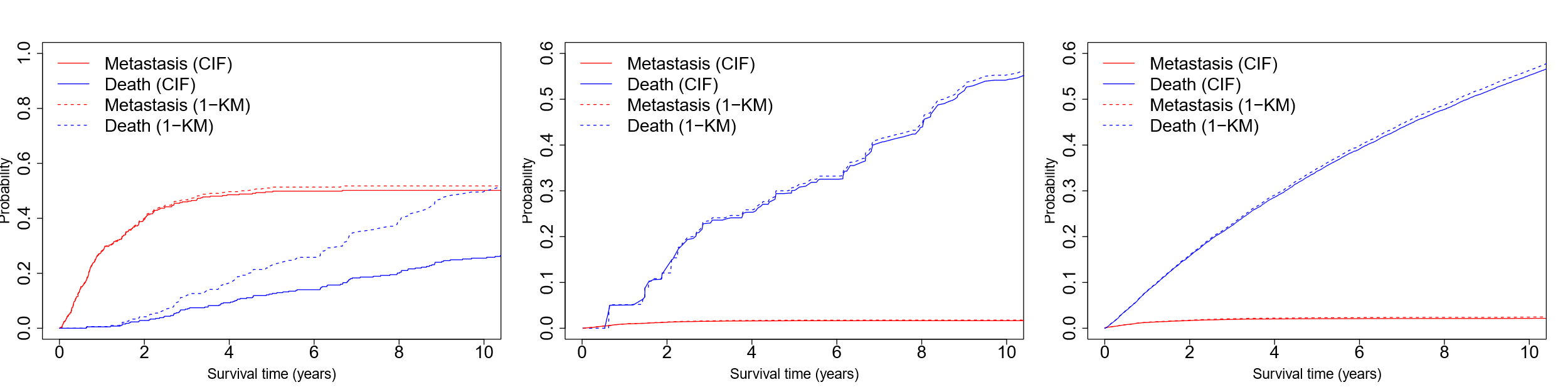


**Supplementary Figure 2** Investigation of the effect of competing events (in particular death due to any cause) on the estimated probability of metastasis. A Fine-Gray model was fitted to the development cohort to estimate the absolute risk of metastasis within five years, taking competing events into account. We compared cumulative incidence estimated with the Fine-Gray model (CIF) and 1- Kaplan-Meier survival estimates (1-KM) for metastatic and death events (red and blue, respectively) using unweighted estimates (left), weighted estimates (middle) and the full cohort (right). For metastatic risk, the difference between CIFs and Kaplan-Meier estimates is small because metastatic events occur substantially earlier than death events. This is observed for all metastatic risk curves, regardless of the sampling weights used. Based on this observation, a Fine-Gray model was not used for the final modelling steps.


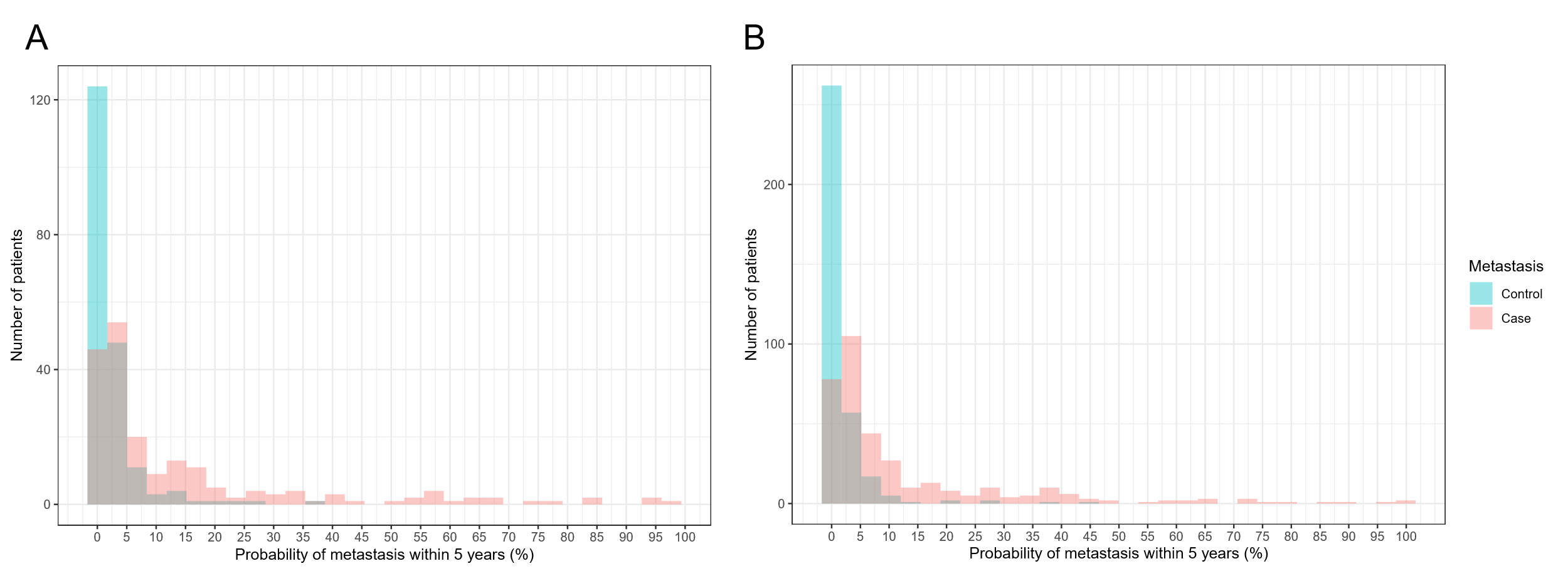


**Supplementary Figure 3** Distribution of risk probabilities estimated by the absolute risk model, for cases and controls in the (A) development cohort (N=390 patients) and (B) validation cohort (N=696 patients).


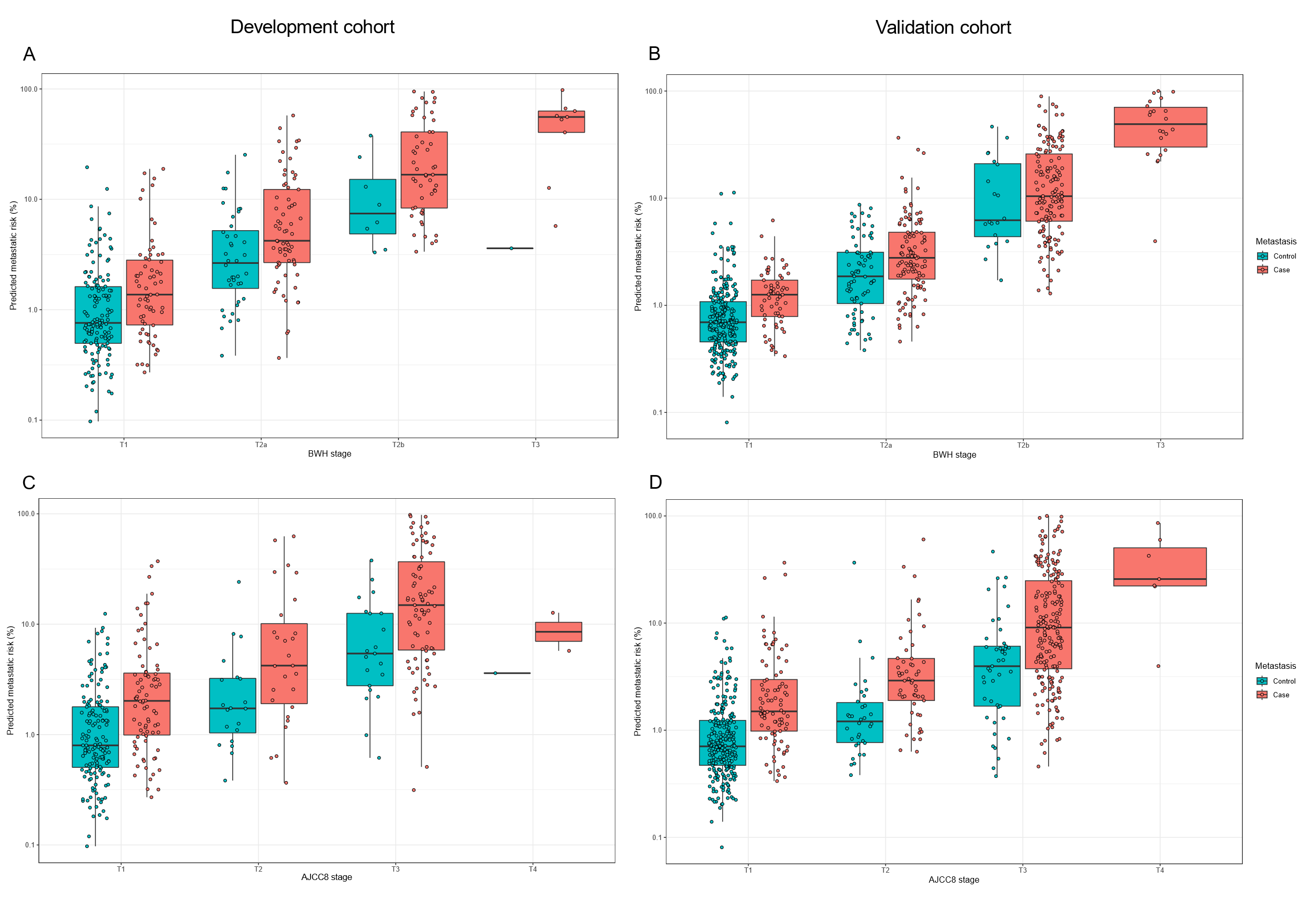


**Supplementary Figure 4** Distribution of estimated metastatic risk probabilities within Brigham and Women’s Hospital (BWH) stages (A and B) and within American Joint Committee on Cancer 8^th^ edition (AJCC8) stages (C and D), for the development cohort (on the left, panels A and C) and the validation cohort (on the right, panels B and D). When some pathological variables were missing, risk probabilities and stages were computed based on multiple imputed data.

**Supplementary Table 1** Predictors considered for the model, how they were measured in the Dutch cohort and how they were recoded for model development. n.a: not applicable, NCR: National Cancer Registry from the Netherlands, HM: Haematological malignancy, OT: Organ transplant, PNI: Perineural invasion, LVI: Lymphovascular invasion

| **Variable** | **Source** | **Description** | **Variable coding** | **Transformation** | **Levels^1^** | **Winsorisation^2^** |
| --- | --- | --- | --- | --- | --- | --- |
| Age | NCR | Patient’s age, in years | Numeric | n.a | n.a | n.a |
| Gender | NCR | Patient’s sex as registered in the NCR | Binary | n.a | Female or male | n.a |
| Number of cSCCs | Manually counted from patient pathology records | Number of cSCCs that the patient has had before the current tumour | Numeric | n.a | n.a | 4 |
| Haematological malignancy | NCR | Indicates if patient has had a haematological malignancy before the diagnosis of the selected cSCC | Binary | Merged into one variable (Immunosuppressed) | Patient has not had any HM nor OT versus patient has had HM or OT | n.a |
| Organ transplant receiver | Linkage with NOTR | Indicates if patient has had an organ transplant before the diagnosis of the selected cSCC | Binary |  |  | n.a |
| Tumour diameter | Pathology record | Macroscopic tumour diameter, as measured by a pathologist, in cm | Numeric | n.a | n.a | 4 |
| Tumour location | Pathology record | Tumour location based on the pathology record | Categorical | Grouped into 3 levels | Trunk and extremities  Face (including ear)  Scalp and neck | n.a |
| Breslow thickness | Re-assessed by a pathologist (AM) | Breslow thickness from the granular layer of the skin to the deepest point, in mm | Numeric | log | n.a | n.a |
| Tissue involvement | Re-assessed by a pathologist (AM) | Information on tissues invaded by the tumour (dermis, subcutaneous fat, muscle, bone, cartilage and other deep structures) | Categorical | Grouped into 3 categories | Tumour invades 1) dermis only 2) the subcutaneous fat only, or 3) beyond the subcutaneous fat. | n.a |
| Differentiation | Re-assessed by a pathologist (AM) | Degree of differentiation according to the simplified Broders' classification which focusses on the percentage of undifferentiated cells/poorly differentiated component: Poor (>75% undifferentiated cells), Moderate (25-75% undifferentiated cells), Good (<25% undifferentiated cells). | Categorical | Grouped into 2 categories | Good/moderate vs Poor | n.a |
| Morphology subgroup | Re-assessed by a pathologist (AM) | Morphology subgroup:  Desmoplastic (>30% desmoplastic component). This subtype is defined as small strands of cSCC surrounded by a prominent desmoplastic stroma. The desmoplastic stroma needs to be extending the area of area of cSCC to be called a ‘desmoplastic component’.  Acantholytic (>30% acantholytic component)  Spindle (>30% acantholytic component) | Binary | 2 categories | No/other subtype vs Acantholytic/Desmoplastic/spindle | n.a |
| Perineural invasion | Pathology records and re-assessed by AM | Perineural invasion (>=0·1 mm) recorded in the pathology records, or identified by AM | Binary | Combined into one variable (Perineural or lymphovascular invasion) | No PNI nor LVI vs PNI or LVI | n.a |
| Lymphovascular invasion | Pathology records and re-assessed by AM | Lymphovascular invasion recorded in the pathology records, or identified by AM | Binary |  |  | n.a |
| Resection margin | Pathology report | Indicates whether excision margins are radical (clear of tumour) or irradical if tumour is still present at the border. | Binary | Excluded due to the following reasons: incomplete resection margins might be a consequence of the surgical procedure performed to excise the cSCC (e.g. Mohs versus standard excision), than on the biology of the cSCC itself. Moreover, it is common practice to perform a re-excision or radiotherapy when excisions are incomplete. Since we only have access to histopathological information, we would not be able to identify those who had adjuvant radiotherapy. This could affect the determination of this variable for many patients with incomplete margins. | | |

^1^For categorical or binary variables, the first category is used as reference in the model.

^2^Winsorisation corresponds to limiting the extreme values of a variable to a maximum value during model development or application. The number indicated in this column corresponds to the maximum value allowed for the corresponding variable. For example, all tumours with a tumour diameter higher than 4 cm were considered to have a tumour diameter of 4 during model development. This should also be done if the model is applied to a tumour whose diameter is higher than 4 cm.

**Supplementary Table 2** Transparent reporting of a multivariable prediction model for individual prognosis or diagnosis (TRIPOD) checklist for prediction model development

| **Section/Topic** | **It** | **Checklist Item** | **Page** |
| --- | --- | --- | --- |
| **Title and abstract** | | | |
| Title | 1 | Identify the study as developing and/or validating a multivariable prediction model, the target population, and the outcome to be predicted. | 1 |
| Abstract | 2 | Provide a summary of objectives, study design, setting, participants, sample size, predictors, outcome, statistical analysis, results, and conclusions. | 1 |
| **Introduction** | | | |
| Background and objectives | 3a | Explain the medical context (including whether diagnostic or prognostic) and rationale for developing or validating the multivariable prediction model, including references to existing models. | 2 |
|  | 3b | Specify the objectives, including whether the study describes the development or validation of the model or both. | 2 |
| **Methods** | | | |
| Source of data | 4a | Describe the study design or source of data (e.g., randomised trial, cohort, or registry data), separately for the development and validation data sets, if applicable. | 3 |
|  | 4b | Specify the key study dates, including start of accrual; end of accrual; and, if applicable, end of follow-up. | 3 |
| Participants | 5a | Specify key elements of the study setting (e.g., primary care, secondary care, general population) including number and location of centres. | 3 |
|  | 5b | Describe eligibility criteria for participants. | 3,S2 |
|  | 5c | Give details of treatments received, if relevant. | - |
| Outcome | 6a | Clearly define the outcome that is predicted by the prediction model, including how and when assessed. | 3 |
|  | 6b | Report any actions to blind assessment of the outcome to be predicted. | 3 |
| Predictors | 7a | Clearly define all predictors used in developing or validating the multivariable prediction model, including how and when they were measured. | S4,S5 |
|  | 7b | Report any actions to blind assessment of predictors for the outcome and other predictors. | - |
| Sample size | 8 | Explain how the study size was arrived at. | 3 |
| Missing data | 9 | Describe how missing data were handled (e.g., complete-case analysis, single imputation, multiple imputation) with details of any imputation method. | 3,S10 |
| Statistical analysis methods | 10a | Describe how predictors were handled in the analyses. | 3,S4,S5 |
|  | 10b | Specify type of model, all model-building procedures (including any predictor selection), and method for internal validation. | 4 |
|  | 10d | Specify all measures used to assess model performance and, if relevant, to compare multiple models. | 4 |
| Risk groups | 11 | Provide details on how risk groups were created, if done. | - |
| **Results** | | | |
| Participants | 13a | Describe the flow of participants through the study, including the number of participants with and without the outcome and, if applicable, a summary of the follow-up time. A diagram may be helpful. | S2 |
|  | 13b | Describe the characteristics of the participants (basic demographics, clinical features, available predictors), including the number of participants with missing data for predictors and outcome. | 5-7 |
| Model development | 14a | Specify the number of participants and outcome events in each analysis. | 5 |
|  | 14b | If done, report the unadjusted association between each candidate predictor and outcome. | - |
| Model specification | 15a | Present the full prediction model to allow predictions for individuals (i.e., all regression coefficients, and model intercept or baseline survival at a given time point). | 7,S12 |
|  | 15b | Explain how to the use the prediction model. | 4,S12 |
| Model performance | 16 | Report performance measures (with CIs) for the prediction model. | 8,9 |
| **Discussion** | | | |
| Limitations | 18 | Discuss any limitations of the study (such as nonrepresentative sample, few events per predictor, missing data). | 5,7-10 |
| Interpretation | 19b | Give an overall interpretation of the results, considering objectives, limitations, and results from similar studies, and other relevant evidence. | 5,7-10 |
| Implications | 20 | Discuss the potential clinical use of the model and implications for future research. | 5,7-10 |
| **Other information** | | | |
| Supplementary information | 21 | Provide information about the availability of supplementary resources, such as study protocol, Web calculator, and data sets. | 4 |
| Funding | 22 | Give the source of funding and the role of the funders for the present study. | 4 |

**Supplementary Table 3** Hazard ratios (before shrinkage) estimated in the development cohort considering complete cases (pairs with no missing data in both cases and control (N=192) and pairs with no missing data in both case and control, after simple estimation of tumour diameter using excision width (N=316)), and in each of the 10 imputed datasets (N=390). The scenario with the estimation of tumour diameter using excision width was performed as follows: if tumour diameter was missing, we assumed that it takes the value of the excision width divided by 1.5225. The factor 1.5225 is the average shrinkage factor observed in the development dataset. It was obtained by dividing the tumour diameter by the width of the excision, for the tumours whose reports contained both the tumour diameter and the width of the excision.

| **Variable** | **Complete cases** | | **Imputation dataset** | | | | | | | | | |
| --- | --- | --- | --- | --- | --- | --- | --- | --- | --- | --- | --- | --- |
|  | **N=192** | **N=316** | **1** | **2** | **3** | **4** | **5** | **6** | **7** | **8** | **9** | **10** |
| Age | 1.32 | 1.33 | 1.37 | 1.33 | 1.38 | 1.36 | 1.36 | 1.36 | 1.37 | 1.36 | 1.34 | 1.32 |
| Gender | 0.95 | 1.64 | 1.86 | 1.80 | 1.86 | 1.77 | 1.88 | 1.82 | 1.92 | 1.81 | 1.83 | 1.95 |
| Number of prior cSCCs | 2.62 | 2.05 | 1.98 | 2.03 | 1.98 | 2.02 | 1.94 | 1.95 | 2.00 | 1.97 | 1.99 | 2.01 |
| Tumour location (Scalp and neck) | 1.16 | 0.71 | 0.38 | 0.41 | 0.45 | 0.46 | 0.45 | 0.46 | 0.38 | 0.46 | 0.42 | 0.36 |
| Tumour location (Face) | 2.57 | 2.11 | 1.69 | 1.88 | 1.75 | 2.17 | 1.86 | 1.77 | 1.91 | 2.02 | 1.93 | 1.62 |
| Tumour diameter | 1.75 | 1.84 | 1.85 | 2.07 | 1.85 | 1.84 | 1.87 | 1.89 | 1.94 | 1.92 | 1.92 | 1.95 |
| Tissue involvement (Subcutaneous fat) | 1.79 | 1.30 | 1.53 | 1.37 | 1.59 | 1.49 | 1.45 | 1.55 | 1.43 | 1.49 | 1.56 | 1.35 |
| Tissue involvement (Beyond subcutaneous fat) | 3.10 | 3.95 | 5.85 | 5.18 | 6.65 | 5.10 | 5.76 | 5.16 | 5.29 | 4.94 | 4.28 | 5.52 |
| Poor differentiation | 6.34 | 4.07 | 4.96 | 4.74 | 4.48 | 4.53 | 4.69 | 4.79 | 4.79 | 4.41 | 4.51 | 5.30 |
| Perineural or lymphovascular invasion | 1.84 | 1.92 | 2.55 | 2.43 | 2.17 | 2.72 | 2.56 | 2.63 | 2.61 | 2.72 | 3.02 | 2.75 |

**Supplementary Table 4** Weighted performance metrics on the development cohort (internal validation with bootstrap, 95% confidence intervals), and on the validation cohort (external validation, bootstrap 95% confidence intervals) for model predictions at 1 year. O/E ratio: Observed to expected events ratio. BWH: Brigham and Women’s Hospital staging system, AJCC8: American Joint Committee on Cancer 8^th^ edition staging system

|  |  | **Development cohort** | | **Validation cohort** | | |
| --- | --- | --- | --- | --- | --- | --- |
|  |  | **C-index** | **Calibration slope** | **C-index** | **Calibration slope** | **O/E ratio** |
| **1-year** | Absolute risk model | 0·84  (0·78-0·88) | 0·94  (0·75-1·12) | 0·86  (0·82-0·89) | 1·05  (0·86-1·24) | 0·88  (0·62-1·14) |
|  | BWH | 0·78  (0·72-0·84) | 0·83  (0·43-0·97) | 0·85  (0·81-0·88) | 0·94  (0·77-1·11) | 0·33  (0·24-0·42) |
|  | AJCC8 | 0·74  (0·66-0·80) | - | 0·83  (0·79-0·86) | - | - |

**Supplementary Table 5** Weighted performance metrics on the validation cohort (external validation, bootstrap 95% confidence intervals) for model predictions at 3 years. Only case-control sets without any missing variables for the absolute risk model/staging systems were considered (N=288). O/E ratio: Observed to expected events ratio. BWH: Brigham and Women’s Hospital staging system, AJCC8: American Joint Committee on Cancer 8^th^ edition staging system

| **N=288** | **1-year** | | | **3-year** | | |
| --- | --- | --- | --- | --- | --- | --- |
|  | **C-index** | **Calibration slope** | **O/E ratio** | **C-index** | **Calibration slope** | **O/E ratio** |
| Absolute risk model | 0·87  (0·82-0·91) | 0·98  (0·76-1.20) | 0·95  (0·6-1·29) | 0·86  (0·81-0·89) | 0·90  (0·65-1·15) | 0·87  (0·56-1·18) |
| BWH | 0·85  (0·8-0·89) | 0·97  (0·73-1·21) | 0·34  (0·19-0·48) | 0·82  (0·78-0·86) | 0·84  (0·63-1·04) | 0·56  (0·31-0·80) |
| AJCC8 | 0·83  (0·77-0·87) | - | - | 0·78  (0·73-0·82) | - | - |

**Supplementary Table 6** Overview of profession and work location of the respondents to the survey (N=53), and their answers regarding metastatic risk cutoff for guiding decisions on follow-up schedule, adjuvant radiotherapy, and systemic treatment of cSCC patients. The survey was sent to 150 members of the Skin Cancer Outcomes (SCOUT) consortium.

| **Variable** | **N** | **N = 53** |
| --- | --- | --- |
| **Profession, n (%)** | 53 |  |
| Dermatologist |  | 20 (38%) |
| Dermatologist, Mohs surgeon |  | 5 (9%) |
| Head and neck surgeon |  | 4 (7%) |
| Medical Oncologist |  | 10 (19%) |
| Radiation Oncologist |  | 11 (21%) |
| Surgeon |  | 2 (4%) |
| Surgical Pathologist |  | 1 (2%) |
| **Work location, n (%)** | 53 |  |
| Europe |  | 12 (23%) |
| North America |  | 34 (64%) |
| Other |  | 7 (13%) |
| **Median threshold for follow-up schedule (%)^1^, (IQR)** | 53 | 10 (5, 20) |
| **Median threshold for adjuvant radiotherapy (%)^2^, (IQR)** | 53 | 20 (10, 20) |
| **Median threshold for adjuvant systemic treatment (%)^3^, (IQR)** | 53 | 30 (20, 40) |
| ^1^ Answer to the question: *Above which 5-year metastatic risk probability would you include a cSCC patient in a follow-up schedule?*  ^2^ Answer to the question: *Above which 5-year metastatic risk probability would you consider discussing adjuvant radiotherapy to the local tumour bed following clear margin surgery with your cSCC patient?*  ^3^ Answer to the question: *Above which 5-year metastatic risk probability would you consider discussing adjuvant systemic treatment with your cSCC patient?*  Abbreviations: IQR : Interquartile range | | |
