# Supplementary material for: Personalised decision making to predict absolute metastatic risk in cutaneous squamous cell carcinoma: development and validation of a clinico-pathological model
Source: eClinicalMedicine. 2023 Aug 19;63:102150. doi: 10.1016/j.eclinm.2023.102150 (PMC10468358; doi:10.1016/j.eclinm.2023.102150)
Supplement: Supplementary Text [file mmc2.docx]

**Supplementary Text**

**Identification of metastasis in the development cohort**

The identification of metastasis in the development cohort was performed as described in the methods section of Tokez et al^1^. Of note, it was not always straightforward to identify the tumour that caused the metastasis in patients with multiple cSCC. In these patients, we have selected the culprit primary cSCC by assessing the following: 1) the proximity of the primary tumour location and the metastatic lymph node (e.g. if a patient had a cSCC on the right arm and another one on the right leg, and they develop a metastasis in the lymph node of the right axilla, we considered the cSCC on the right arm to be the most likely culprit); 2) the time interval between the cSCC and the detection of the metastasis (e.g. a primary cSCC that occurred within 2 years before the metastasis is more likely to be the cause of the metastasis compared to a cSCC that occurred more than 5-10 years before) and 3) (high) risk factors of the potential culprit cSCC. Since we were studying risk factors, criterion 3 was more of an extra criterion to further support assignments based on criteria 1 and 2. We excluded the cases in which it was difficult to determine which cSCC was the culprit with these criteria (n=5 out of 267), as shown in the consort diagram of Supplementary Figure 1.

**Multiple imputation**

Missing values were assumed to be missing at random and were imputed using multiple imputation using chained equations (*mice* R package^2^ (v3.13.0)). All variables in Supplementary Table 1 as well as the metastasis outcome and the Nelson-Aalen estimate of the cumulative hazard were used as potential predictors in the imputation model. The type of material (biopsy or excision) and tumour width as reported in the pathology reports were used as auxiliary variables together with other pathological variables scored by the pathologist in this cohort for other research (depth of invasion, mitotic rate, tumour budding, solar elastosis, keratinocytic dysplasia, peritumoral infiltration, keratinisation). Ten imputed datasets were created. The convergence of the mice algorithm was checked by visualizing the convergence plots and 50 iterations were used. Coefficients were pooled using Rubin’s rules.^3^

Multiple imputation was performed in a similar way in the validation cohort, using 10 imputed datasets. The variables used in the imputation were slightly different than in the development cohort because some were not available in this cohort. Therefore, we used all variables in Supplementary Table 1 for imputation, as well as the metastasis outcome and the Nelson-Aalen estimate of the cumulative hazard. Ethnicity and deprivation were used as auxiliary variables.

Performance metrics were pooled across multiple imputed datasets using the *pool_auc* function of the *psfmi* R package (v1.0.0).^4^

**Computation of sampling weights**

In weighted Cox regression models, weights are assigned to subjects to compensate for the difference in the ratio of cases to controls between nested case-control (NCC) datasets and full cohorts. The sampling weights adjust the contribution of controls to the model and take matching into account. Weights can be estimated using different methods.^5^ In our model, we computed the weight of subject $i$($w_{i}$) as the inverse of the probability of the subject being included in the NCC dataset ($w_{i}=\frac{1}{p_{i}})$. Since all cases in the full cohort were sampled, their sampling probability is 1 and consequently their weights are also 1. For controls, the sampling probability was estimated using Kaplan-Meier type of estimators, taking matching into account. Specifically, the sampling probability ($p_{i})$ of control $i$ being sampled from the full cohort was computed as follows:

$$p_{i}=1-\prod_{j} 1-\frac{m_{ij}}{n_{j}\left( t_{j} \right)}I (Control i could be sampled for case j)$$

Where:

- $m_{ij}$ is the number of cSCC tumours of control $i$ that can be sampled for case $j$;
- I is an indicator variable that accounts for matching: it takes the value 1 if the control i has a cSCC with longer follow-up than that of case j and both were diagnosed in the same pathology lab, and 0 otherwise
- $n_{j}\left( t_{j} \right)$ the number of cSCCs tumours at risk at time $t_{j}$, which could be matched to case $j$ and belonged to non-metastatic patients.

Of note, patients with suspected metastasis were excluded as potential controls (see Supplementary Figure 1A).

Sampling weights were computed in a similar way for the validation cohort, with one difference for the weights of the cases. As shown in Supplementary Figure 1B, cases with incomplete cSCC records were not included in the NCC cohort (N=84). This means that the sampling probability of the cases was actually $p_{i}=\frac{432-84}{432}=0.806$, instead of 1. The case weights should account for this.^6^ Therefore, cases in the validation cohort were assigned a weight of $w_{i}= \frac{1}{0.806}=1.24$.

**Mathematical formula of the absolute risk model**

Step 1: Calculate the linear predictor:

$$\boldsymbol{linear predictor}=-1.86+0.25*\left( \boldsymbol{Age}-7.47 \right)+0.51*\boldsymbol{Gender}+0.57*(\boldsymbol{Ncscc}-0.29)+0.54*(\boldsymbol{Tdiam}-1.20)-0.72*\boldsymbol{ScNeck}+0.52*\boldsymbol{Face}+0.33*\boldsymbol{SC}+1.40*\boldsymbol{BSC}+1.30*\boldsymbol{Diff}+0.80*\boldsymbol{PLI}$$

Step 2: Calculate the metastatic risk probability:

$$\boldsymbol{p}_{\boldsymbol{5}\boldsymbol{y mets}}\boldsymbol{=}1-{0.973}^{exp(linear predictor)}$$

$$where:$$

$\boldsymbol{Age}$: Patient’s age, in decades (e.g. 85 years should be introduced in the formula of step 1 as 8.5)

$\boldsymbol{Gender}$***:*** Male=1, Female=0

$\boldsymbol{Ncscc}$*:* Number of prior cSCCs (excluding current cSCC): 0 to 4 (>4 is considered 4)

***Tdiam:*** Tumour diameter in cm: if tumour is larger than 4 cm, *Tdiam* is considered to be 4

***ScNeck:*** Tumour is located on the scalp or neck: Scalp/neck=1, Other areas = 0

***Face:*** Tumour is located on the face: Face=1, Other areas = 0

$\boldsymbol{SC}$***:*** Tumour invades subcutaneous fat, but does not go beyond subcutaneous fat =1, Tumour stays in the dermis or goes beyond subcutaneous fat = 0

***BSC:*** Tumour invades beyond subcutaneous fat =1, Tumour does not invade beyond subcutaneous fat = 0

$\boldsymbol{Diff}$**:** Differentiation grade: Poor =1, Good/moderate =0

$\boldsymbol{PLI}$**:** Presence of perineural or lymphovascular invasion: Present =1, Absent =0

**References**

1. Tokez S, Wakkee M, Kan W, Venables ZC, Mooyaart AL, Louwman M, et al. Cumulative incidence and disease-specific survival of metastatic cutaneous squamous cell carcinoma: A nationwide cancer registry study. *J Am Acad Dermatol*. 2022;**86**(2):331–8.

2. van Buuren S, Groothuis-Oudshoorn K. mice: Multivariate imputation by chained equations in R. *J Stat Softw*. 2011;**45**(3):1–67.

3. van Buuren S. Flexible Imputation of Missing Data. CRC Press. 2012. 1–299 p.

4. Heymans MW. psfmi: Prediction Model Pooling, Selection and Performance Evaluation Across Multiply Imputed Datasets. https://mwheymans.github.io/psfmi/. 2021.

5. Støer NC, Samuelsen SO. MultipleNCC: Inverse probability weighting of nested case-control data. *R J*. 2016;**8**(2):5–18.

6. Zhou QM, Wang X, Zheng Y, Cai T. New weighting methods when cases are only a subset of events in a nested case-control study. *Biometrical J*. 2022;**64**(7):1240–59.
