# Supplementary figures and images for: Personalised decision making to predict absolute metastatic risk in cutaneous squamous cell carcinoma: development and validation of a clinico-pathological model
Source: eClinicalMedicine. 2023 Aug 19;63:102150. doi: 10.1016/j.eclinm.2023.102150 (PMC10468358; doi:10.1016/j.eclinm.2023.102150)

A

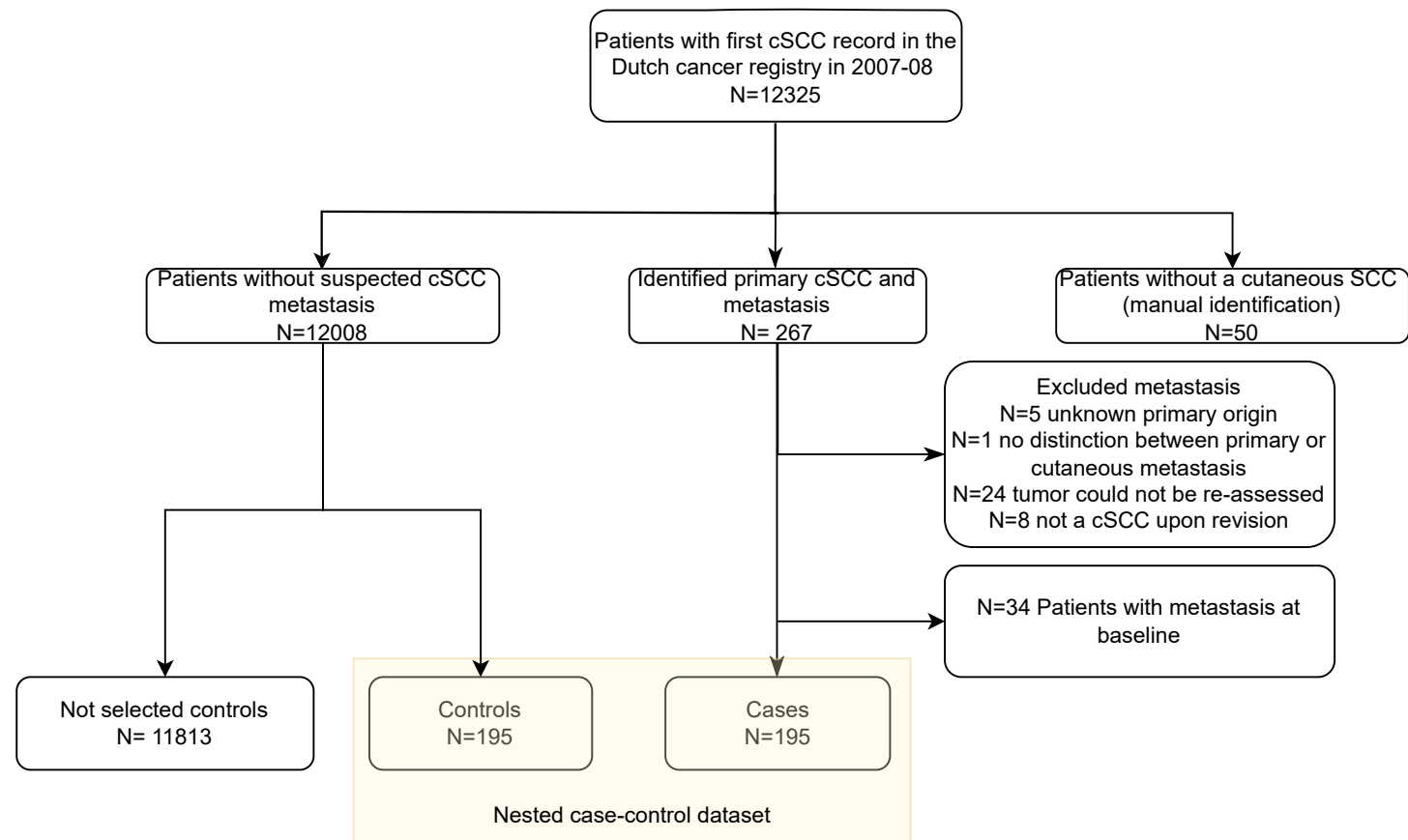

B

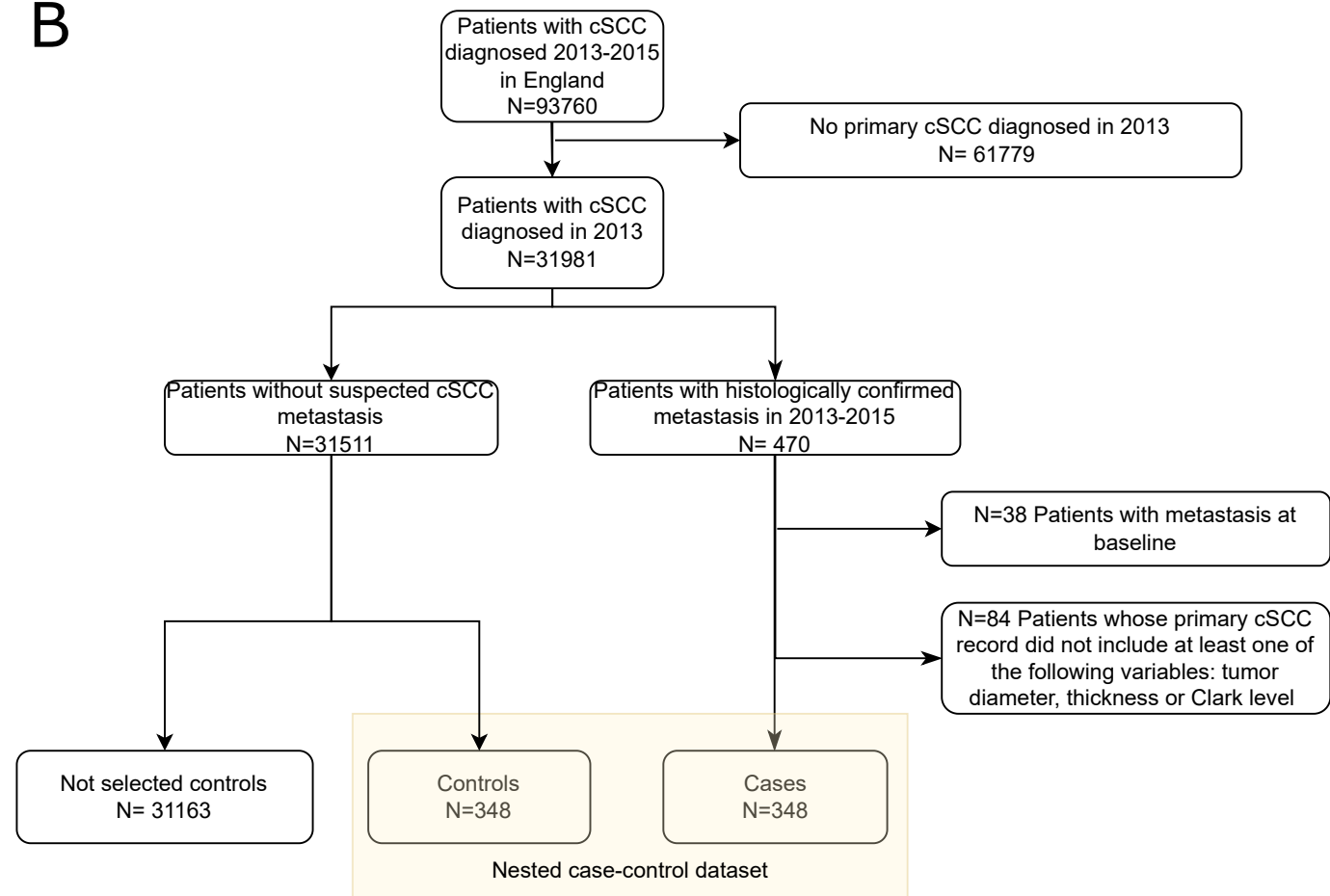

Supplement: Supplementary Figure S1 [file mmc3.pdf]

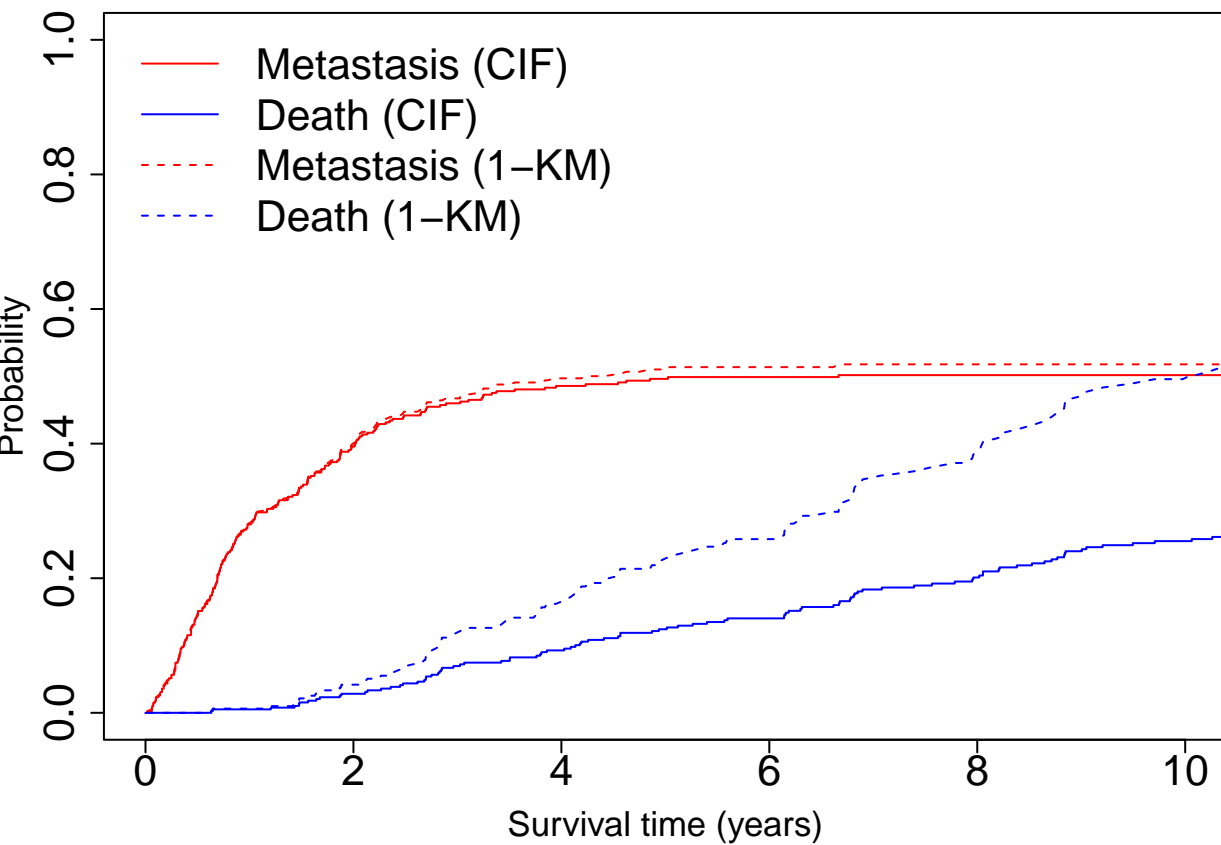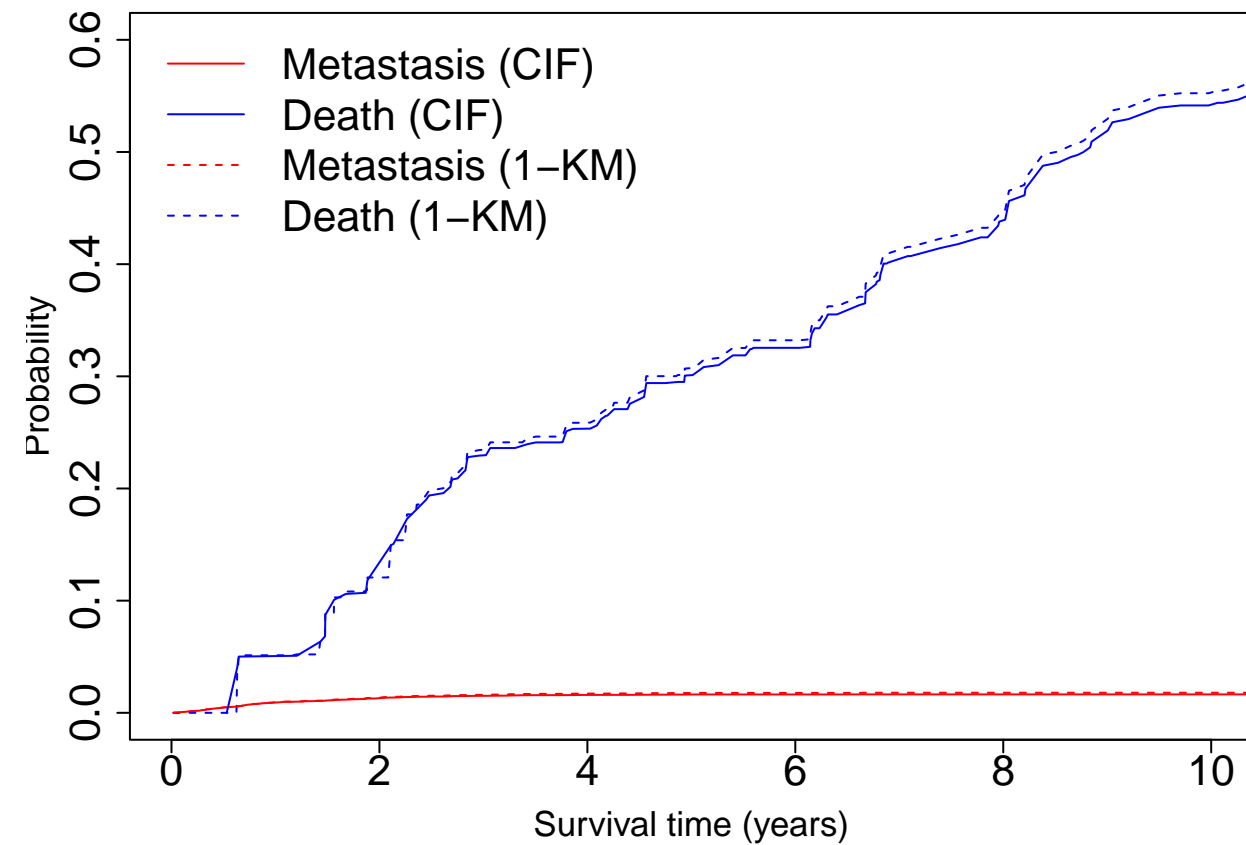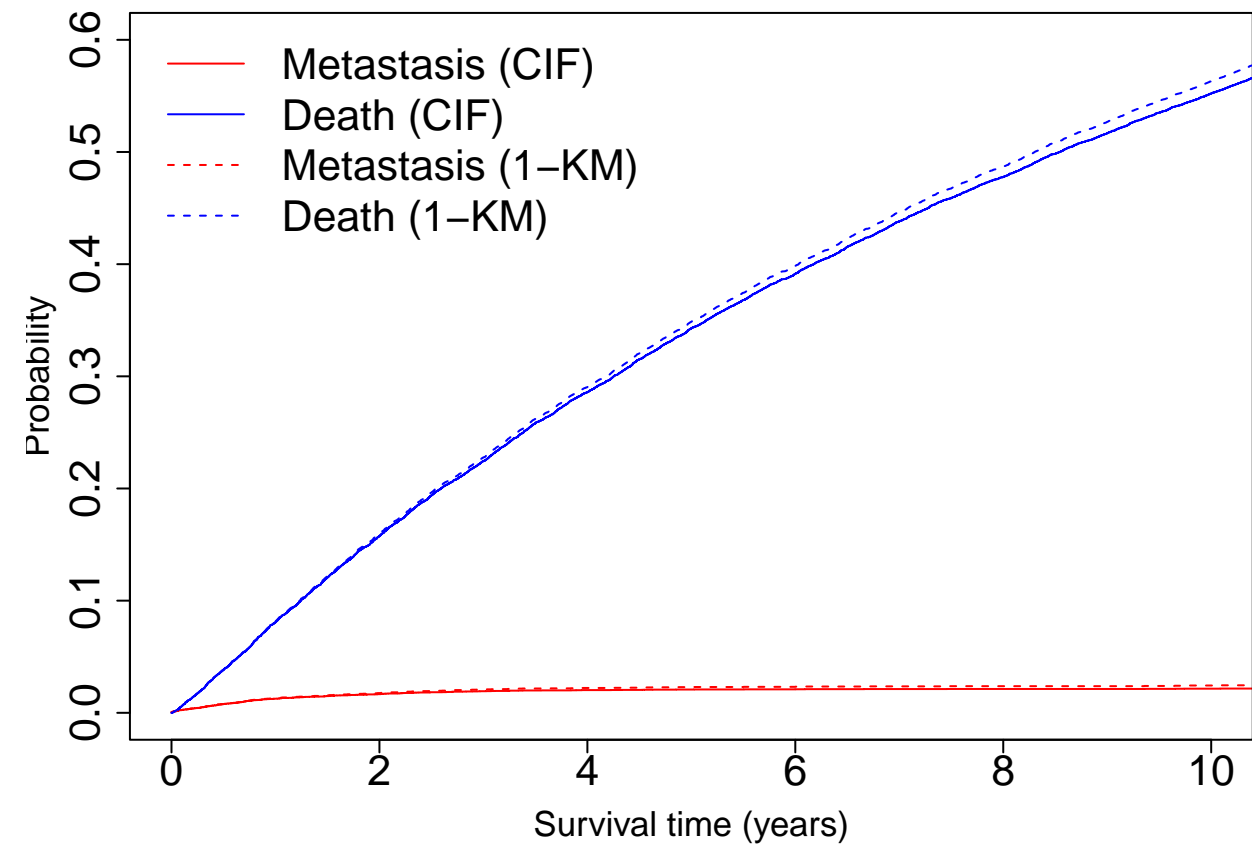

Supplement: Supplementary Figure S2 [file mmc4.pdf]

A

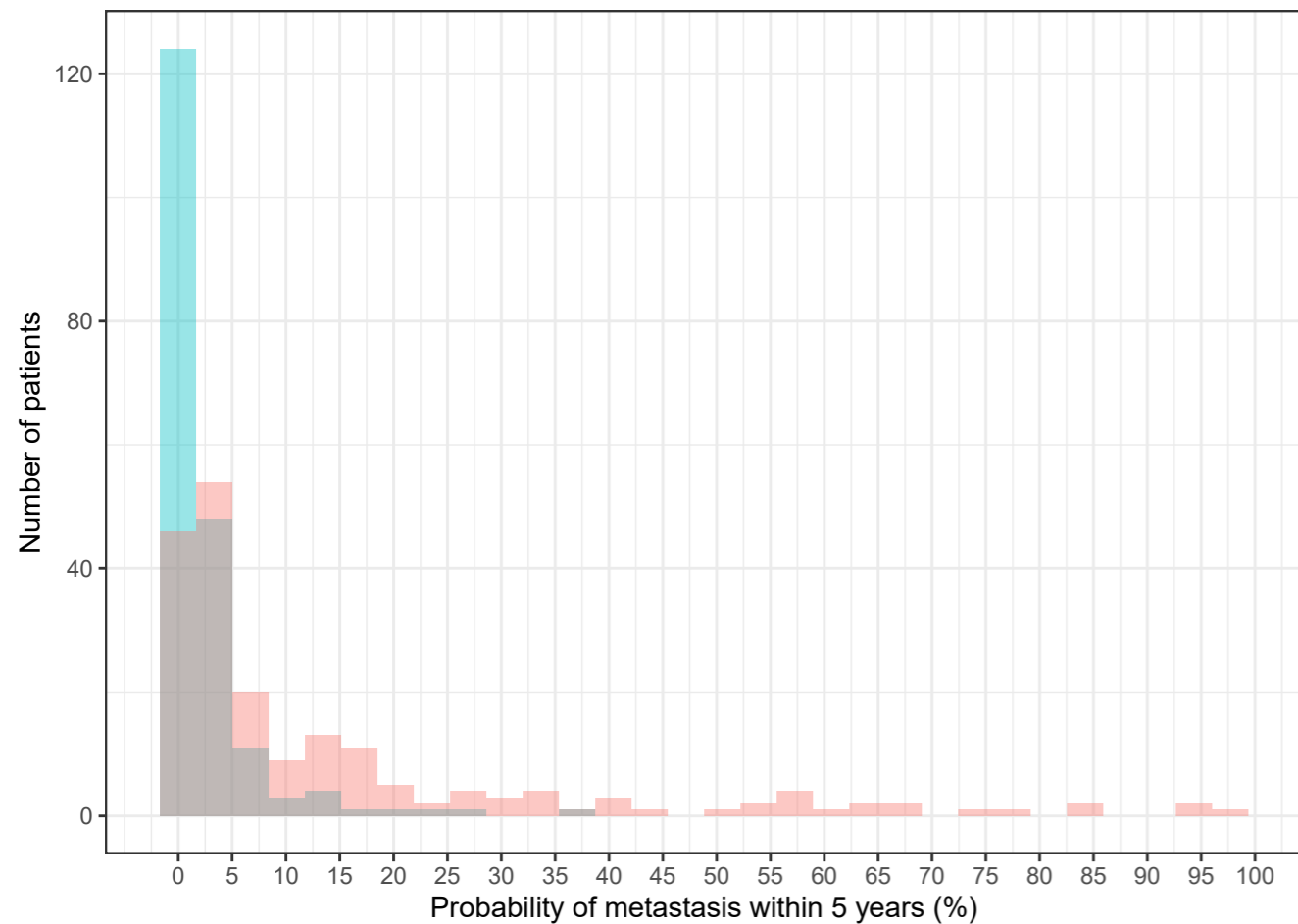

B

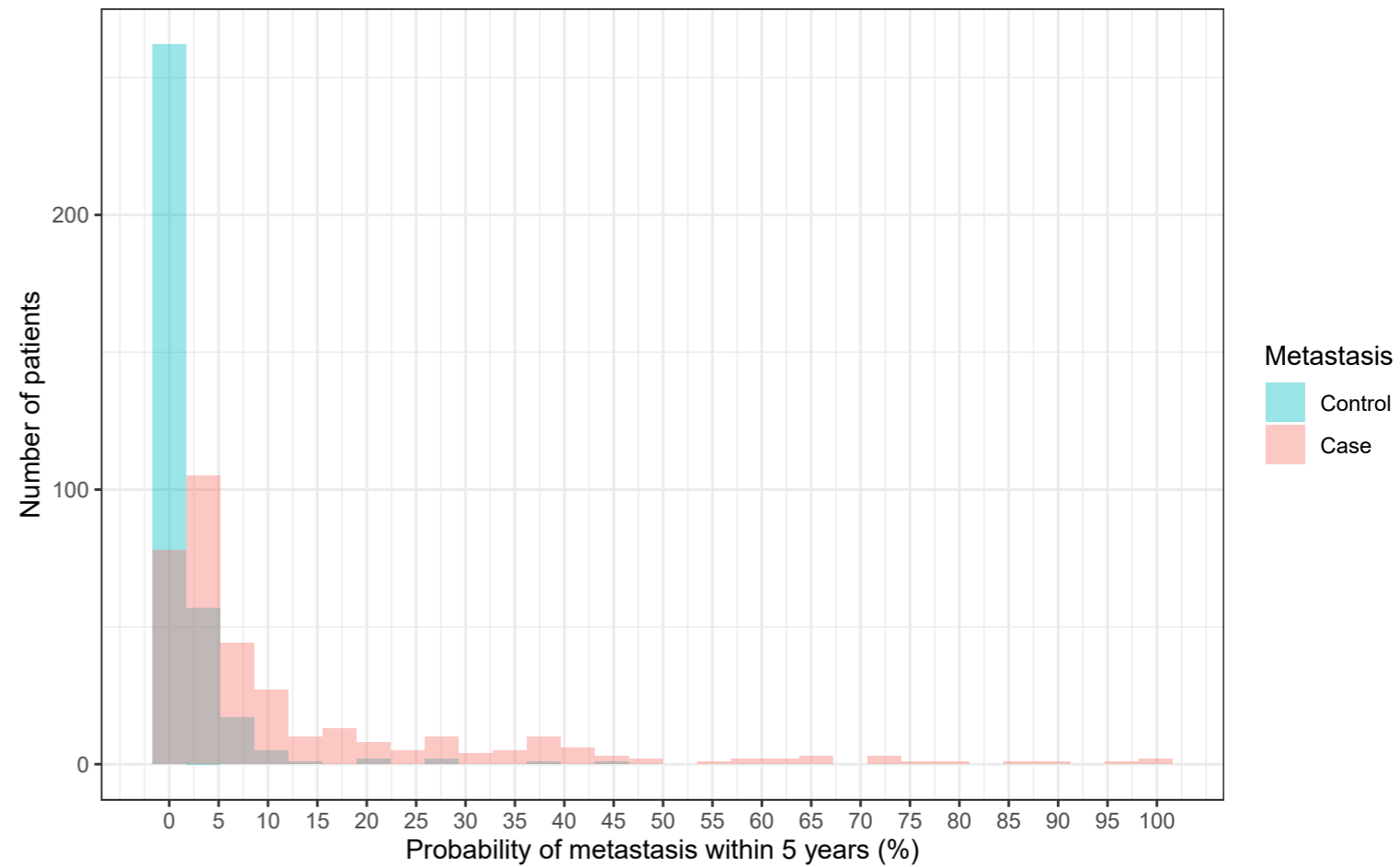

Supplement: Supplementary Figure S3 [file mmc5.pdf]

Development cohort

A

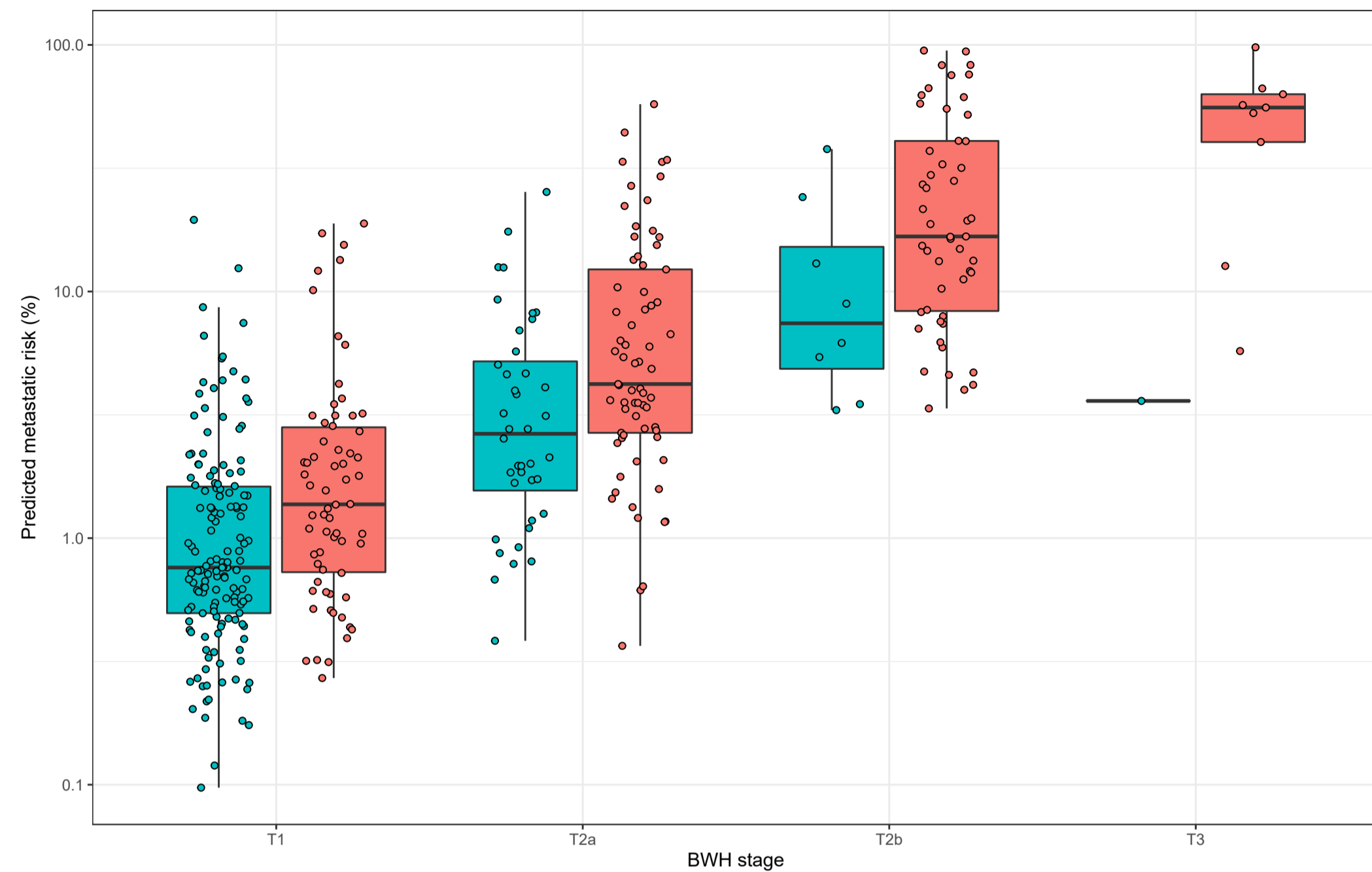

Validation cohort

B

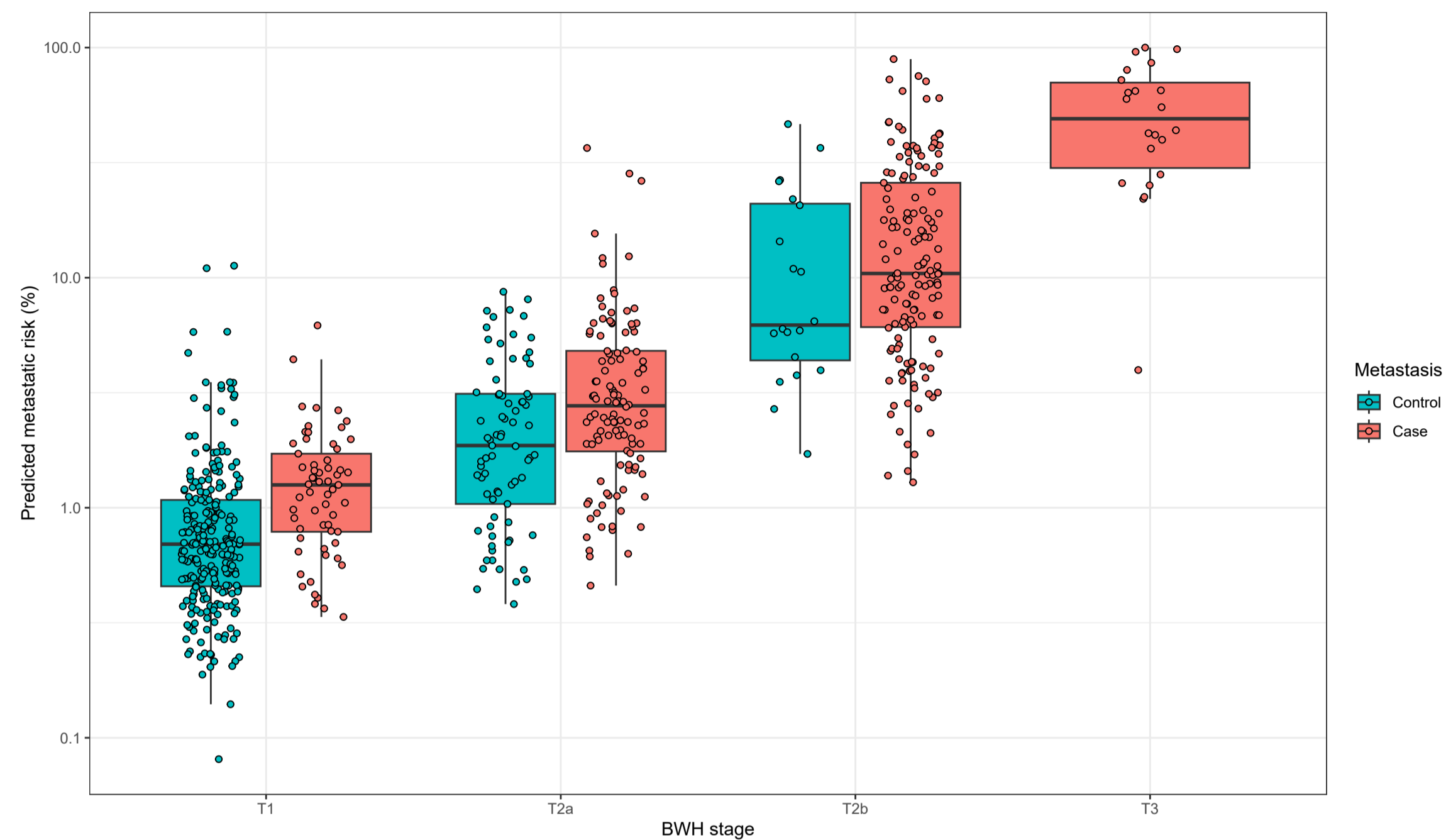

C

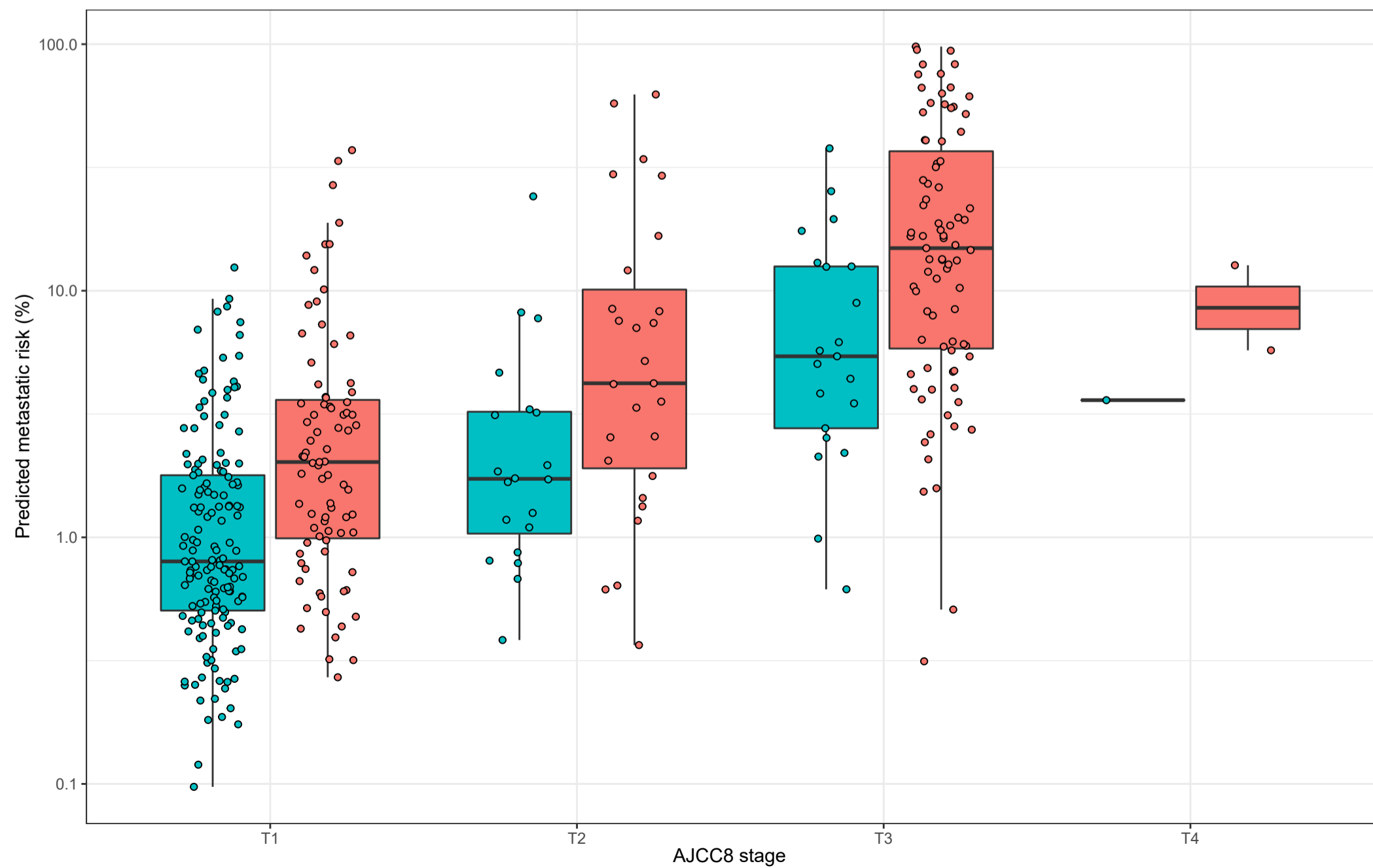

D

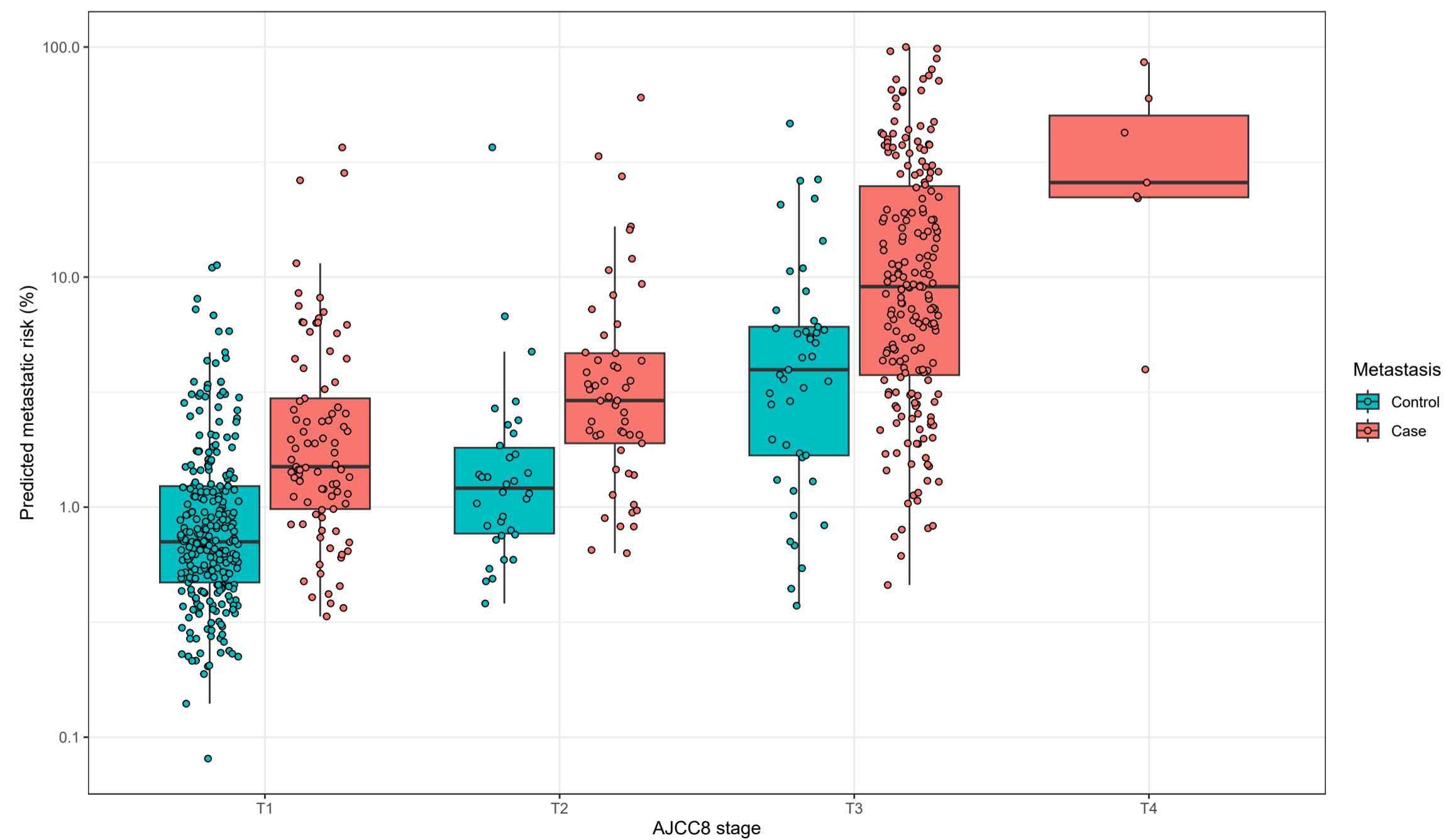

Supplement: Supplementary Figure S4 [file mmc6.pdf]
